# Healing of Preterm Ruptured Fetal Membranes

**DOI:** 10.1038/s41598-017-13296-1

**Published:** 2017-10-13

**Authors:** Haruta Mogami, Annavarapu Hari Kishore, Yucel Akgul, R. Ann Word

**Affiliations:** 1 The Cecil H. and Ida Green Center for Reproductive Biological Sciences, Department of Obstetrics and Gynecology, University of Texas Southwestern Medical Center, Dallas, Texas USA; 2 Department of Plastic Surgery, University of Texas Southwestern Medical Center, Dallas, Texas USA

## Abstract

Preterm premature rupture of membrane (pPROM) is associated with 30–40% of preterm births. Infection is considered a leading cause of pPROM due to increased levels of proinflammatory cytokines in amniotic fluid. Only 30%, however, are positive for microbial organisms by amniotic fluid culture. Interestingly, in some pregnancies complicated by preterm premature rupture of membranes (pPROM), membranes heal spontaneously and pregnancy continues until term. Here, we investigated mechanisms of amnion healing. Using a preclinical mouse model, we found that small ruptures of the fetal membrane closed within 72 h whereas healing of large ruptures was only 40%. Small rupture induced transient upregulation of cytokines whereas large ruptures elicited sustained upregulation of proinflammatory cytokines in the fetal membranes. Fetal macrophages from amniotic fluid were recruited to the wounded amnion where macrophage adhesion molecules were highly expressed. Recruited macrophages released limited and well-localized amounts of IL-1β and TNF which facilitated epithelial-mesenchymal transition (EMT) and epithelial cell migration. Arg1 + macrophages dominated within 24 h. Migration and healing of the amnion mesenchymal compartment, however, remained compromised. These findings provide novel insights regarding unique healing mechanisms of amnion.

## Introduction

Preterm labor is the leading cause of perinatal morbidity and mortality^1^. Preterm premature rupture of membrane (pPROM) is defined as the rupture of membrane occurring before 37 weeks of gestation, which is associated with 30–40% of preterm deliveries and occurs in approximately 1–3% of all pregnancies^2^. Amniotic fluid cultures indicate that 30% of pPROM are positive for microbial organisms^3^. Infection-related pPROM requires immediate intervention (delivery) for fear of infection to fetus such as fetal inflammatory syndrome, which is a risk of severe neonatal morbidity with respiratory distress syndrome, neonatal sepsis, pneumonia, chronic lung disease, necrotizing enterocolitis, intraventricular hemorrhage, and cerebral palsy^4^, as well as maternal complication such as sepsis. On the other hand, the majority of pPROM cases are unrelated to infection but may be associated with smoking, low body mass-index, maternal stress, and intrauterine bleeding. In addition, iatrogenic pPROM is caused by amniocentesis or fetoscopy, and accidental rupture of membrane during surgery such as cervical cerclage. It is generally thought that rupture of membrane is irreversible event because most women with pPROM begin labor spontaneously within several days. A small proportion, however, remain undelivered^2^ with spontaneous sealing of the membranes^5^. The mechanisms that promote healing of the fetal membranes are unknown.

Macrophages play an important role in wound healing^6,7^. Macrophages phagocytose pathologic organisms and matrix debris, removing necrotic tissue. Macrophage also releases various growth factors and cytokines at the wound site, reconstituting the wound site by forming new blood vessels and regulating fibroblast recruitment. Although gradations occur in macrophage classifications, in general, macrophages differentiate into two phenotypes: Classical interferon-γ (IFN-γ)-activated macrophages (M1 macrophages) by T helper 1 (Th1)-type responses play a role of cellular immunity to infections whereas alternative activated macrophage (M2 macrophages) activated by Th2-type cytokines IL-4 and IL-13 are important in allergic reactions, parasitic infections, fetal tolerance, and tissue repair^8,9^. M2 macrophages exhibit potent anti-inflammatory activity and serve important roles in wound healing. Furthermore, M2-like macrophages antagonize proinflammatory M1-macrophage responses^10^.

Fetal membranes are comprised of amnion, chorion, and decidua. Amnion is the primary load-bearing structure of the fetal membranes^1^ and is composed of two cell types: superficial epithelial cells and underlying mesenchymal cells. Interstitial collagens (types I, III, and V) are produced by amnion mesenchymal cells to maintain the mechanical integrity of the amnion. Protected inside these durable fetal membranes, amniotic fluid serves a defensive role as an innate immune system^11^, and a reservoir for fetal macrophages^12^. Maternal macrophages are enriched in maternal-derived decidua^13^.

Here we investigated the mechanism of wound healing using sterile rupture of the fetal membranes in preclinical mouse models (small and large ruptures). Whereas small ruptures of the amnion closed by 72 h, > 50% of large ruptures remained open. Macrophages from amniotic fluid were recruited to the wounded amnion where macrophage adhesion molecules were highly expressed. Initially, recruited macrophages released limited and well-localized amounts of IL-1β and TNF. These proinflammatory cytokines facilitated epithelial-mesenchymal transition (EMT) in human amnion epithelial cells, and EMT was also observed in the healing amnion of mice. Arg1-positive macrophages were dominant within 24 h. Using this mouse fetal membrane rupture model, we conclude that fetal membranes heal by unique mechanisms with compromise of amnion mesenchymal cell migration in large ruptures.

## Results

### Preclinical mouse model of sterile ruptured membrane

**Figure 1:**
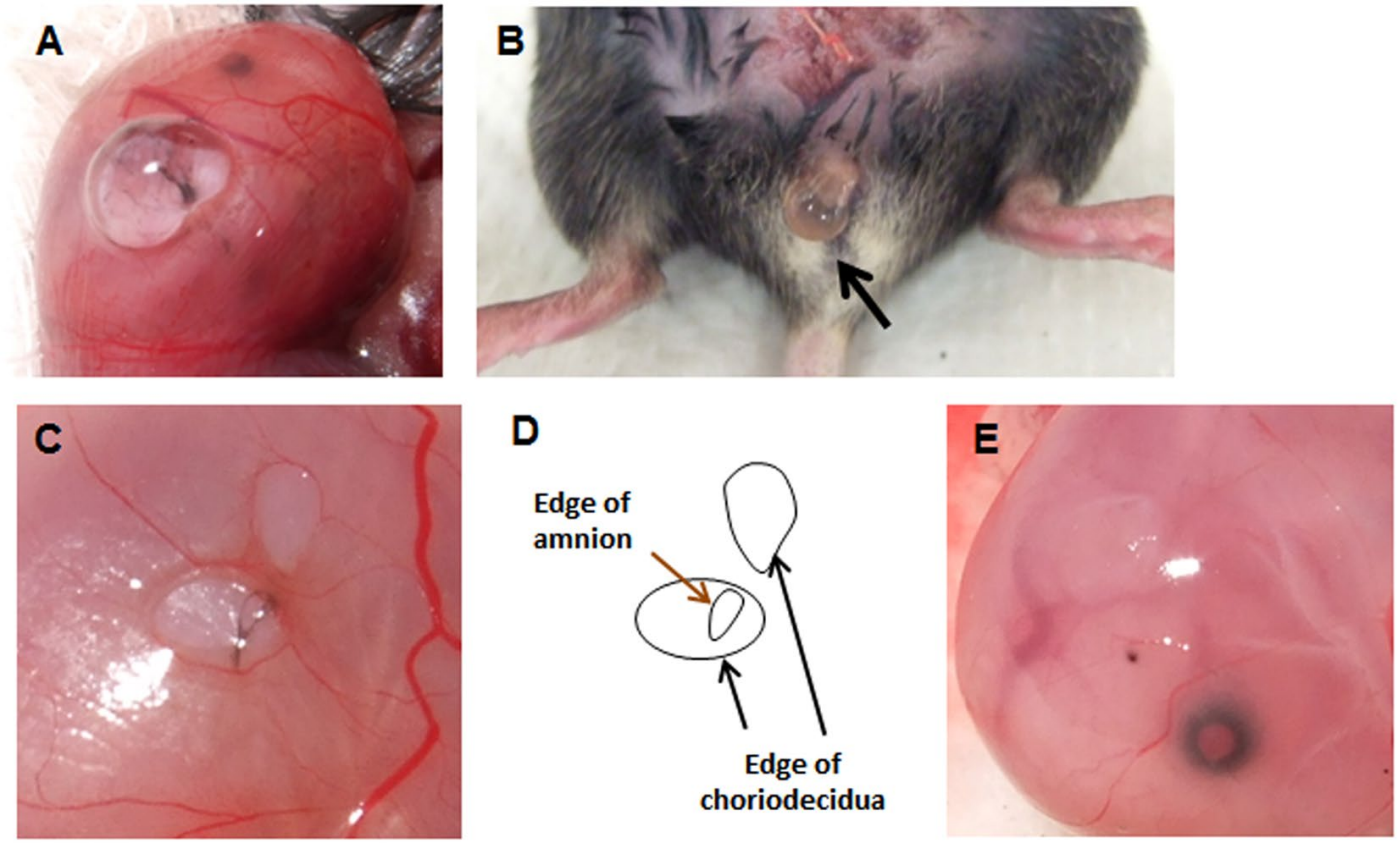
Mouse model of sterile ruptured membranes with 26 gauge (ø 0.47 mm) needle. Leakage of amniotic fluid after puncture through fetal membrane (**A**) and into vagina through cervix (**B**). (**C**) Ruptured fetal membrane 24 h after rupture with 20 ga needle. Myometrium has been removed to visualize membrane defects. Note that edge of ruptured amnion was stained with black ink. (**D**) Schematic of panel C illustrating choriodecidual and amnion defects. (**E**) Completely healed amnion and choriodecidua 72 h after rupture with 26 ga needle.

**Figure 2:**
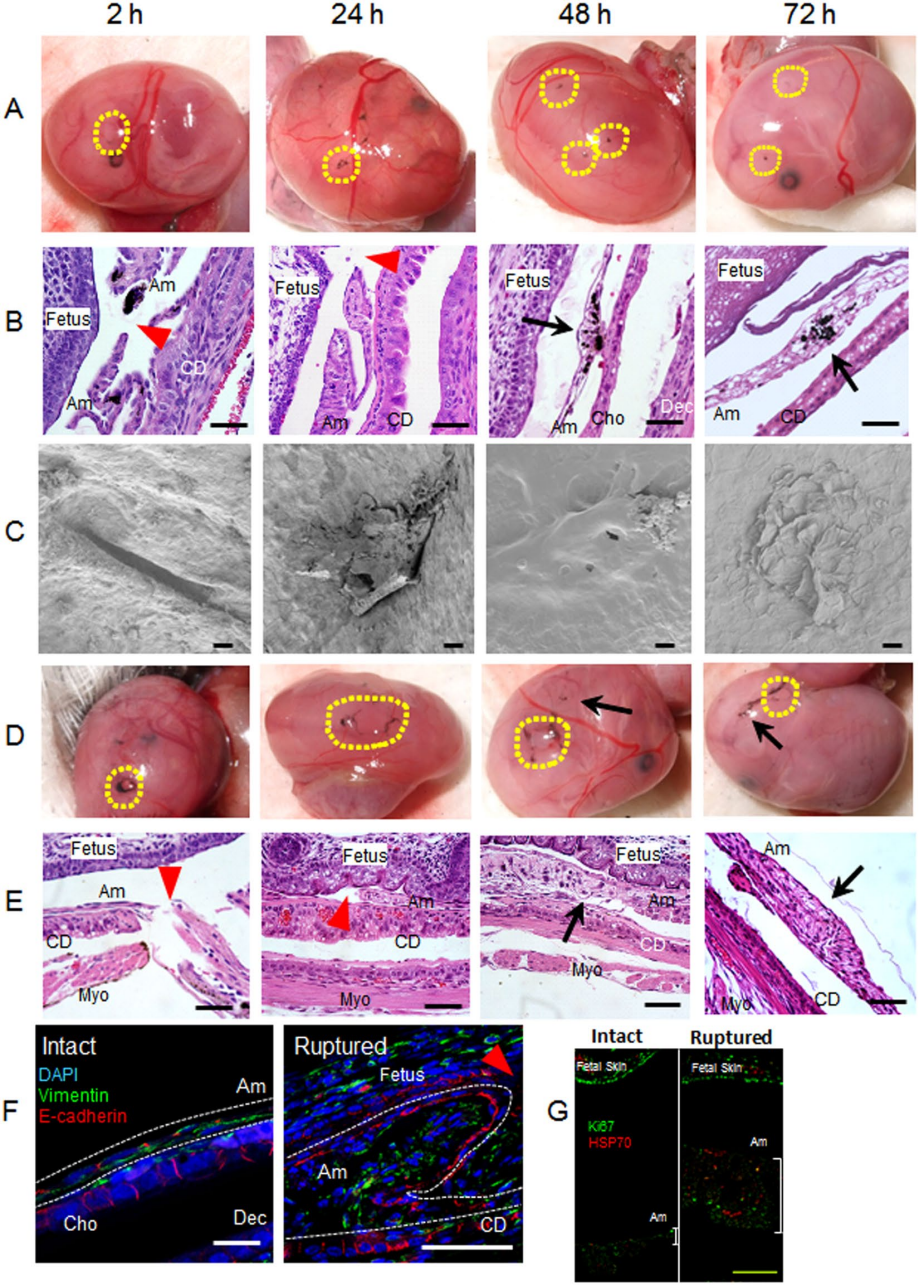
Preclinical mouse model of sterile ruptured membranes. (**A**–**F**) Time course of amnion healing. (**A**) Macroscopic images of ruptured fetal membranes with 26 G needle. Ruptured site was stained with India ink generating black staining at the center of circles outlined in yellow. (**B**) Hematoxylin-Eosin (H&E) staining of ruptured fetal membranes by 26 G needle (x 400). Whole gestational sac including myometrium, fetal membranes, and fetus was fixed and syectioned at the site of injury. **Am**, amnion; **Cho**, chorion; **Dec**, decidua; **CD**, choriodecidua; **Myo**, myometrium. Red arrowhead indicates the defect site of amnion and black arrow indicates healed site. Note that ruptured site was stained with black India Ink. Bars, 50 µm. (**C**) Images of scanning electron microscopy (SEM). After removal of fetus and placenta, images of fetal membranes were captured from inside the gestational sac. Hence, the surface shown here is the epithelial layer of amnion. Bars, 20 µm. (**D**–**E**) Sterile ruptured membrane with 20 gauge (ø 0.91 mm) needle. (**D**) Macroscopic images of ruptured fetal membranes at indicated time after 20 G rupture. Yellow dashed lines encircle ruptured sites stained with black India Ink. Yellow dashed lines encircle open puncture wounds (not healed) whereas arrow indicates closed healing sites. (**E**) H&E staining of fetal membranes after 20 G rupture at indicated time. Bars, 50 µm. (**F**) Immunofluorescence staining for vimentin (green), E-cadherin (red), and DAPI (blue) of fetal membranes from intact site on embryonic d15 (**Intact**) or edge of healing amnion 24 h after 20 G rupture (**Ruptured**). Amnion is circled by white dash lines. Bars, 50 µm. (**G**) Immunolocalization of HSP70 (red) and Ki67 (green) in intact (non-ruptured) and healing ruptured membranes 24 h after 20 G puncture. Note proximity of thickened amnion to fetal skin compared with intact nonruptured membrane. Bar, 100 µm.

A mouse model of sterile rupture of the fetal membranes was generated using puncture of the amniotic membrane with 26 or 20 G needles (outer diameter 0.47 or 0.91 mm) at 15 embryonic day (ED). By puncture, amniotic fluid leaked through myometrium (Fig. 1A) and in the vagina through the cervix (Fig. 1B). After removal of myometrium, ruptured edge of choriodecidua was readily visible (Fig. 1C,D), and long axis of rupture was measured. Edge of amnion was usually stained with black ink (Fig. 1C) thereby distinguished from choriodecidua. Long axis of amnion rupture was also measured (Fig. 1D). For cases in which rupture of amnion was unclear, choriodecidua was gently slid with cotton swab to clarify edge of ruptured amnion. If amnion was healed completely, stain of amnion looked like a dot (Fig. 1E). Two h after rupture, perforations were macroscopically visible (Fig. 2A, 2 h). At 24 h, almost half of injuries were still visible, but perforation diameter decreased significantly. Rupture was closed after 48 and 72 h (Fig. 2A). Histologic analysis of the rupture site in transverse cross-section revealed that membrane structure was interrupted 2 h after rupture (Fig. 2B, 2 h). After 24 h, amnion thickness increased at the edge of injury (Fig. 2B, 24 h). In our model, 26 G-induced perforations of amnion were virtually closed by a monolayer of epithelial cells covering stratified mesenchymal cells within 48–72 h (Fig. 2B, 24–72 h).

Scanning electron microscopy (SEM) was used to clarify the ultrastructure of healing fetal membranes. Images were taken from inside the gestational sac, so that images reflect the inner surface of amnion lining the gestational sac. Interestingly, collapsed perforations with surgically-precise edges were readily observed 2 h after rupture (Fig. 2C, 2 h). At 24 h, amnion cell migration and matrix deposition was noted in the rupture site, generating a plug- or flap-like structure (Fig. 2C, 24 h). At 48 and 72 h, the rupture site was closed with thickening and bulging, presumably reflecting migration of mesenchymal cells and matrix deposition as seen by light microscopy (Fig. 2C, 48 h and 72 h). In addition, the ruptured site appeared to be covered by amnion epithelial cells (Fig. 2C, 48 h and 72 h, higher resolution images are shown in Fig. S1A,B).

Next, we compared rapid complete closure of 26 G punctures with larger 20 G ruptures (outer diameter, 0.91 mm). In contrast to virtually 100% healing of small ruptures, almost half of 20 G rupture sites remained open even after 72 h (Fig. 2D). Histologic analysis of the rupture sites revealed accumulation of amnion mesenchymal cells (Fig. 2E, 48 h and 72 h). SEM images of the large rupture model were not possible due to adherence of the ruptured membrane to the fetus from severe loss of amniotic fluid. Thus, after fixation, the membrane was fragile and destroyed during removal of the fetus from the gestational sac. To study the dynamics of healing of 20 G rupture sites, immunofluorescence studies were conducted with markers of epithelium and mesenchyme. In intact fetal membranes, the epithelial cell marker E-cadherin was predominantly localized at cell-cell junctions of amnion epithelial cells and chorion (Fig. 2F). Staining was highly localized and organized at regular intervals between flattened epithelial cells. Vimentin-positive amnion mesenchymal cells were localized to 1-2 cell layers beneath the epithelial lining (Fig. 2F, intact), and fibroblasts in the chorion were also vimentin-positive (Fig. 2F, intact). In the injured epithelium, E-cadherin staining was localized diffusely throughout the cell membrane within 24 h (Fig. 2F, ruptured). Vimentin-positive amnion mesenchymal cells were prominent at the rupture site resulting in a thickened edge of healing amnion (Fig. 2F, ruptured).

To determine if this thickened edge of healing amnion was due to proliferation, Ki67 immunostaining was colocalized with HSP70 to ensure correct localization of the injury site (Fig. 2G). In non-ruptured membranes, HSP70 was punctate and sparsely distributed. In the ruptured membrane after 24 h, HSP70 was more intense and widely distributed throughout the thickened amnion which is closer to the fetal skin due to loss of amniotic fluid (Fig. 2F). Interestingly, Ki67 was not increased in the injured amnion relative to intact membranes and less than the robust Ki67 staining observed in proliferating fetal skin (Fig. 2G).

**Figure 3:**
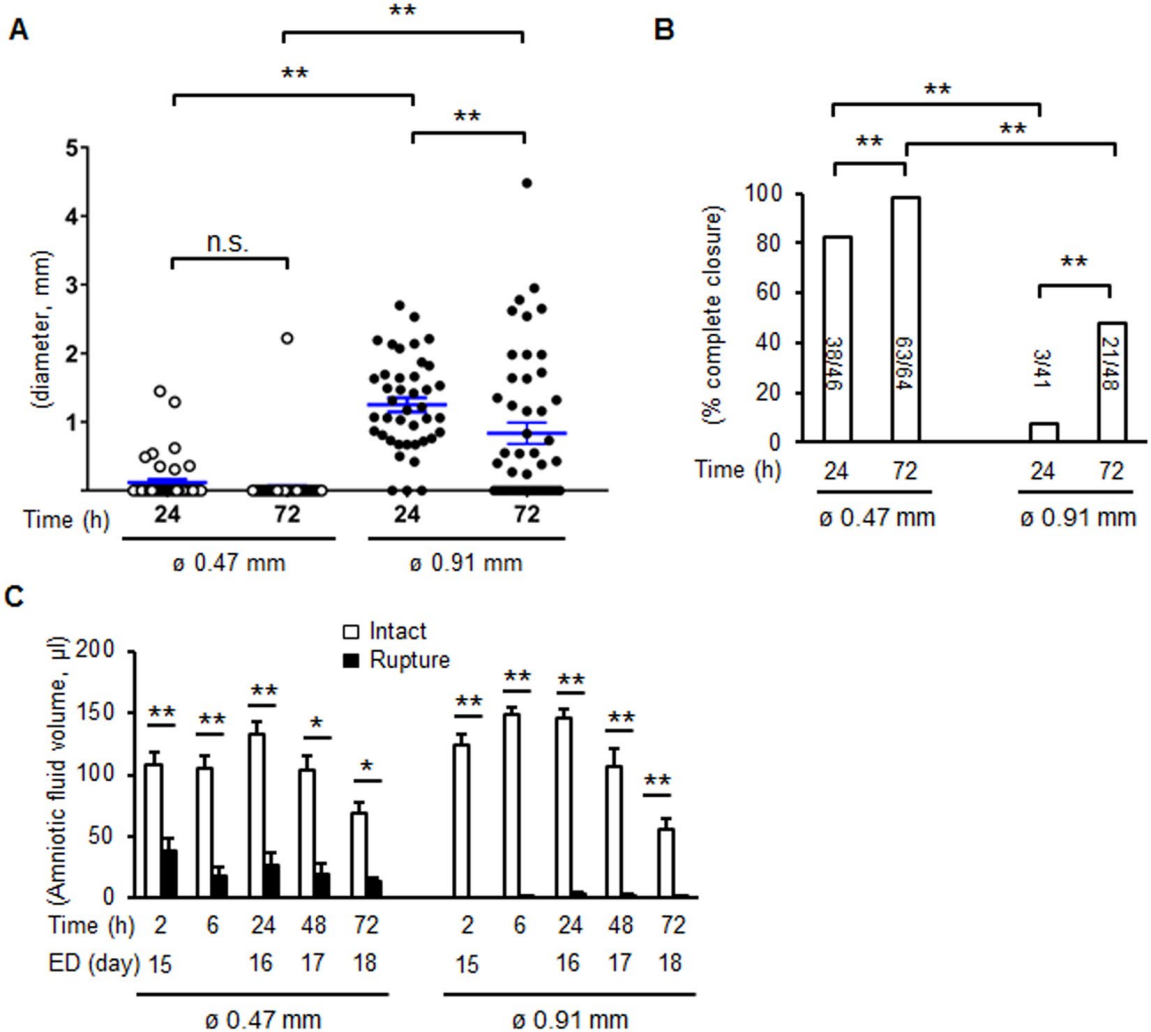
Healing rate and perforation diameters of amnion after membrane puncture. (**A**) Diameter of ruptured membrane sites in amnion with 26 G (ø 0.47 mm) or 20 G needle (ø 0.91 mm) at 24 and 72 h of puncture. Each symbol represents one rupture. Blue bar indicates mean and SEM. **P < 0.01, ANOVA. (**B**) Percent complete closure of ruptured amnion at 24 and 72 h. Number of completely closed ruptures/total ruptures is shown as the bar. *P < 0.05, *P < 0.01, χ^2^. n = 41–64 punctures from 12–17 fetal membranes of 3–7 pregnant mice in each group. (**C**) Amniotic fluid volume after rupture with a 26 G (ø 0.47 mm) or 20 G needle (ø 0.91 mm). ED, embryonic day. Values were compared to the volume of intact gestational sac at each time point. Error bars represent SEM. n = 6–20 gestational sacs from 3–7 pregnant mice in each group. *P < 0.05, **P < 0.01.

The average diameter of small ruptures was 0.12 mm at 24 h and 0.03 mm at 72 h whereas in large ruptures, diameters of 1.25 mm at 24 h (increased significantly compared with small rupture at 24 h) decreased to 0.82 mm at 72 h (*P* < 0.01 compared with small rupture at 72 h) (Fig. 3A). In the small rupture model, average complete closure of amnion was 83% at 24 h and 98% at 72 h (Fig. 3B). In the large rupture model, however, closure rates were only 7% at 24 h and 48% at 72 h (Fig. 3B). In choriodecidua, the average diameter of small rupture was 0.31 mm at 24 h and 0.24 mm at 72 h (statistically not significant), whereas in large rupture, healing was remarkably delayed: 1.38 mm at 24 h (*P* < 0.01 compared with small rupture) and 1.29 mm at 72 h (*P* < 0.01 compared with small rupture, Fig. S2A). Closure of choriodecidua was impaired significantly relative to amnion with 61% and 78% at 24 and 72 h, respectively in small rupture model (Fig. S2B). In the large rupture model, the choriodecidua did not heal at 24 h (no closures) and only 16% at 72 h (Fig. S2B).

At the time of puncture, significant amounts of amniotic fluid leaked from the gestational sac with increased losses in the large rupture model (Fig. 3C). Interestingly, although puncture wounds closed in the small rupture group, amniotic fluid volume did not recover at 48 and 72 h. Despite significant decreases in amniotic fluid, intrauterine fetal survival rates were not decreased after small rupture (>86%) but compromised somewhat by large rupture at 72 h (from 100 to 82%, P < 0.05, χ^2^). This increased fetal loss appeared to be prolapse of the umbilical cord through the ruptured site. The majority of fetuses appeared healthy with normal fetal growth despite oligohydramnios (fetal weight: 1.06 g in intact, 1.00 g in ø 0.47 mm, and 1.01 g in ø 0.91 mm, n = 4–6 in each group). Further, markers of lung alveolar epithelium differentiation were similar in fetal lung from intact and ruptured membranes on d18.5 (Supplementary Table 2).

### Unique gene expression profiles of wound healing in the injured amnion

**Figure 4:**
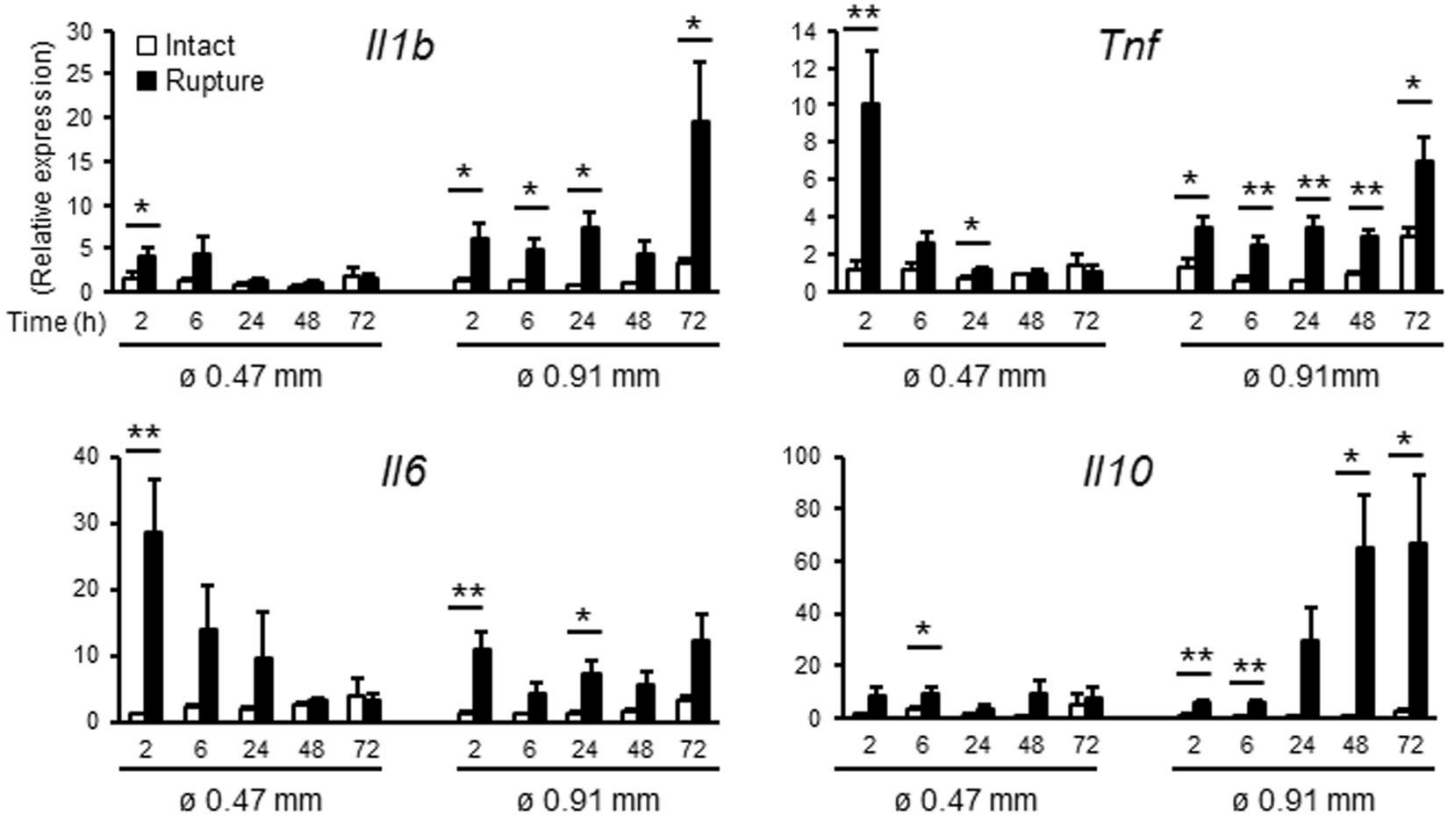
Rupture of fetal membranes alters inflammatory gene expression *in vivo*. Expression of *Il1b*, *Tnf*, *Il6*, and *Il10* in intact or ruptured (ø 0.47 mm: 26 G needle with 8 punctures/sac, and ø 0.91 mm: 20 G needle with 4 punctures/sac) fetal membranes. Relative mRNA expression of each gene normalized to that of β_2_ microglobulin was compared to levels of intact membrane at 2 h. For statistical analysis, gene expression in ruptured membrane was compared with that of intact membrane at each time point. Error bars represent SEM. n = 5–6 fetal membranes from 5-6 pregnant mice in each group. *P < 0.05, **P < 0.01.

Classically, adult wound healing is comprised of four steps: (1) hemostasis, (2) inflammation, (3) proliferation and (4) remodeling^6,7,14^. Injured vessels play a major role in this process from the early process of clot formation, immune cell recruitment, and release of growth factors from neoangiogenesis. The unique nature of the avascular amnion comprised of fetal cells suggests that basic wound-healing mechanisms must differ in fetal membranes. To investigate these mechanisms, gene expression profiles related to wound healing were quantified in fetal membranes with or without injury (Fig. 4). Using fetal membranes *in vivo* (samples that include amnion, chorion, decidua, and all cells attached to membrane), mRNA levels of proinflammatory cytokines, *interleukin-1β* (*Il1b*), *tumor necrosis factor* (*Tnf*), and *Il6* increased within 2 h after small puncture, returning to normal levels by 24 h. In contrast, after large rupture, relatively high levels of *Il1b*, *Tnf*, and *Il6* mRNA persisted until 72 h. Interestingly, the anti-inflammatory cytokine, *Il10*, was also remarkably upregulated in ruptured membranes, especially after large rupture (60-fold compared with 8-fold for *Tnf*, Fig. 4).

Growth factors play an important role in wound healing of many adult tissues. In the healing of fetal membranes, however, mRNA levels of these growth factors (epidermal growth factor, *Egf*; basic fibroblast growth factor, *Bfgf)* or *Fgf2*; transforming growth factors, *Tgfb1* and 3; vascular endothelial growth factor, *Vegf*; and insulin-like growth factors, *Igf1* and 2) were not induced by sterile rupture (with the exception of a slight increase in *Tgfb2* after large rupture) (Fig. S3). Since these growth factors are largely involved in angiogenesis, it is reasonable that they were not regulated in the avascular amnion. Even TGF-β2 did not stimulate migration of either epithelial or mesenchymal cells of amnion (data not shown). Overall, the results illustrate that amnion does not heal by the same mechanisms of other vascularized tissues.

**Figure 5:**
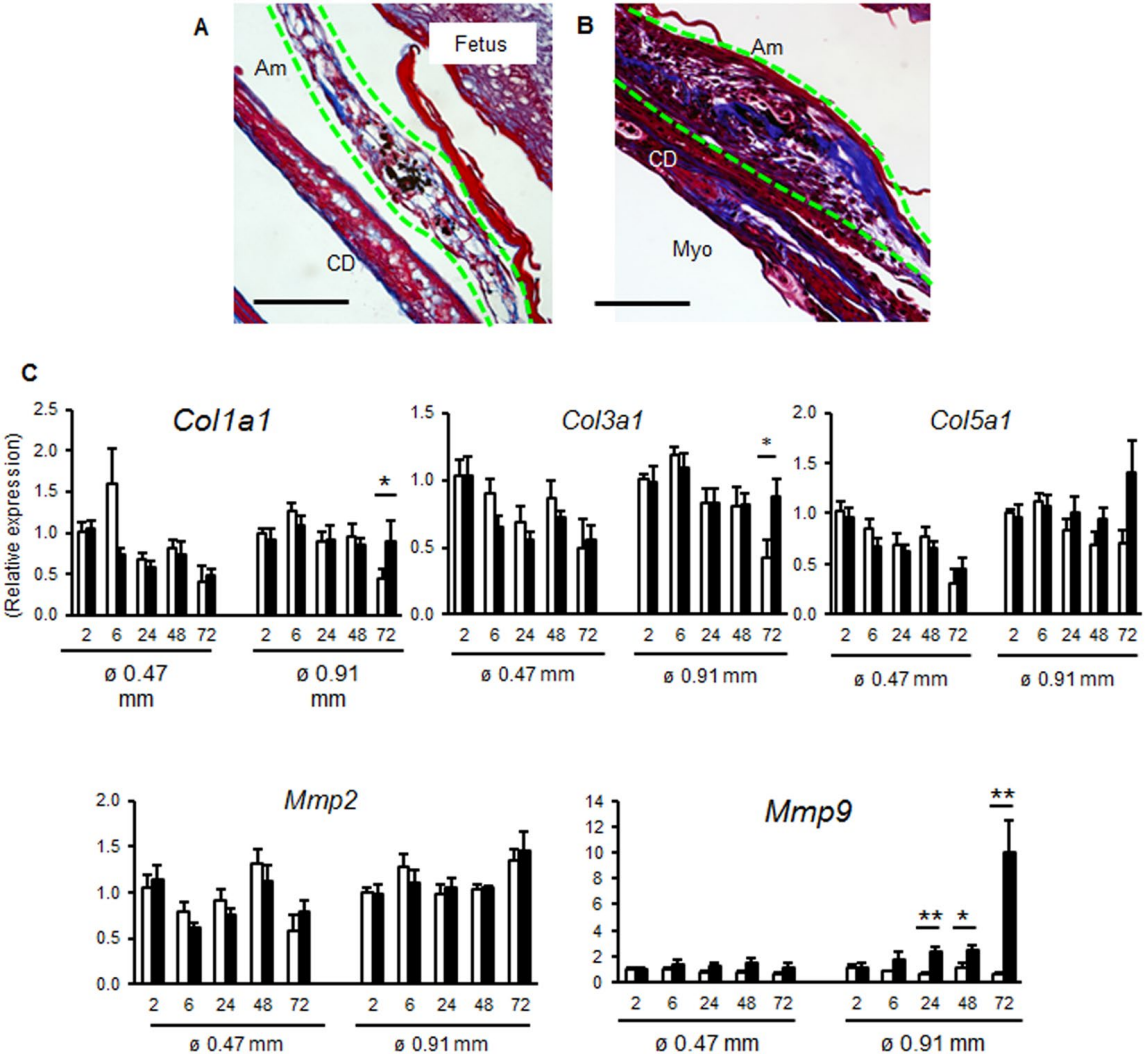
Collagen deposition in fetal membranes after small or large rupture. Trichrome staining of ruptured fetal membrane 72 h after puncture with 26 G (**A**) or 20 G (**B**) needle. Amnion is indicated by green dashed lines. Bar, 50 µm. (C) Expression of *Col1a1, Col3a1, Col5a1, Mmp2*, and *Mmp9* in intact or ruptured (ø 0.47 mm: 26 G needle with 8 punctures/sac, and ø 0.91 mm: 20 G needle with 4 punctures/sac) fetal membranes. Relative mRNA expression of each gene normalized to that of β_2_ microglobulin was compared to levels of intact membrane at 2 h. For statistical analysis, gene expression in ruptured membrane was compared with that of intact membrane at each time point. Error bars represent SEM. n = 5–6 fetal membranes from 5–6 pregnant mice in each group. *P < 0.05, **P < 0.01.

Collagen matrix is remodeled in the later stage of wound healing. Hence, collagen formation at the healing site was compared using trichrome staining at 72 h and qPCR of collagen mRNA after 26 or 20 G rupture. After 26 G rupture, collagen fibers were thin forming a loose supportive network of the healed amnion (Fig. 5A) and mRNA levels of types 1, 3 and 5 collagen were not increased (Fig. 5C). In contrast, collagen deposition was dense with large deposits in the thickened healing sight after 20 G rupture (Fig. 5B). Further, type 1 and 3 collagen mRNA increased at 72 h (Fig. 5C). *MMP9* mRNA was also upregulated from 24 h in the large rupture (Fig. 5C). Collectively, active collagen synthesis and matrix remodeling occurred in the healing membrane after large rupture. The lack of increased Ki67 at the wound site, together with vimentin-positive cells within the thickened edge, suggests that healing of the wound is due to migration, EMT, and matrix deposition.

### Recruitment of amniotic fluid macrophages in ruptured amnion

**Figure 6:**
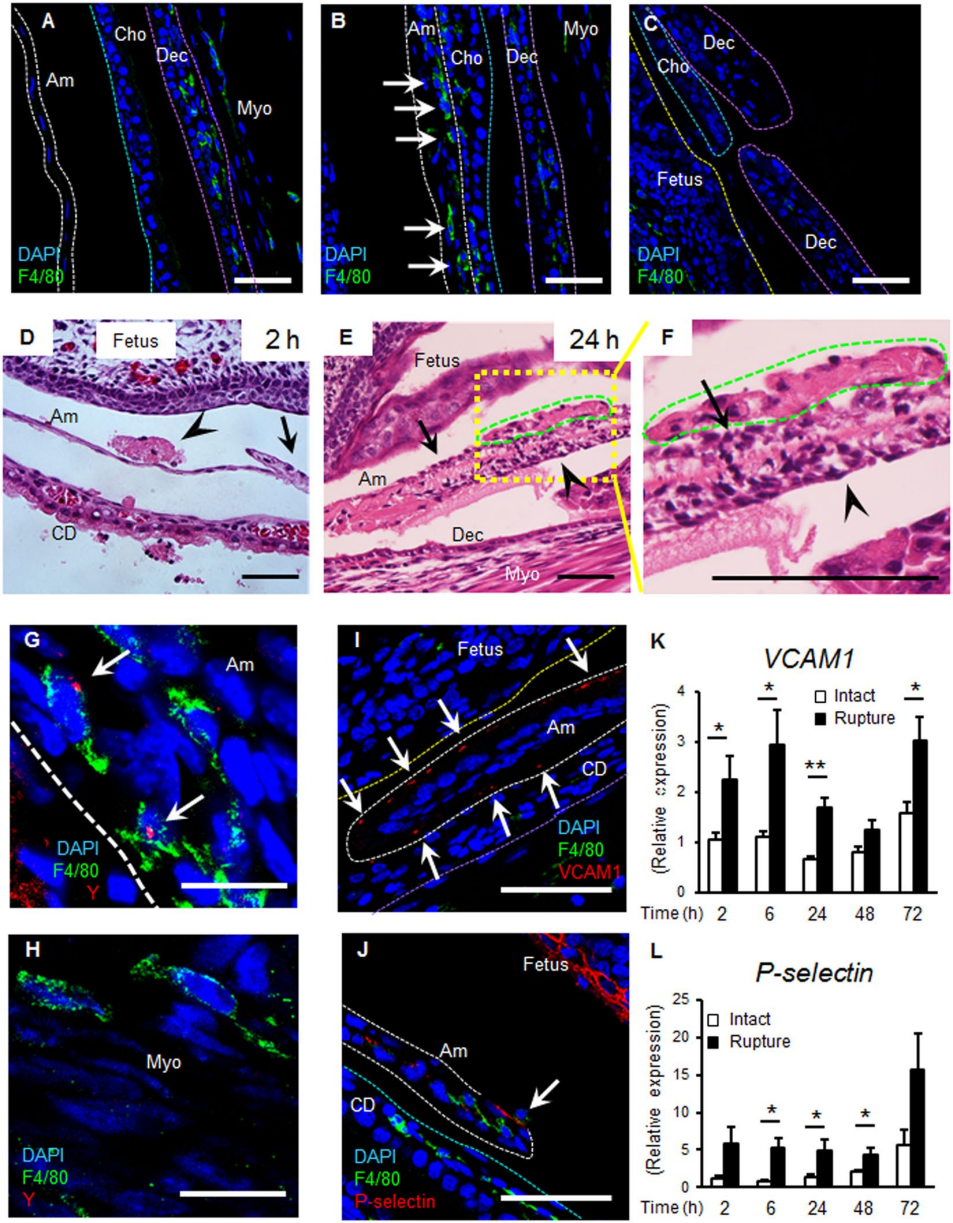
Macrophages and adhesion molecules at rupture site. (**A**–**C**) Immunostaining for F4/80 (green) and DAPI (blue) of membrane 24 h after rupture by 20 G needle. Dashed lines indicate amnion (**Am**, white), surface of fetal skin (yellow), chorion (**Cho**, light blue), and decidua (**Dec**, purple). **Myo**, myometrium. (**A**) Intact fetal membrane. (**B**) Macrophages within healing amnion (arrows). (**C**) Ruptured edge of chorio-decidua. Bars, 50 µm. (**D**–**F**) H&E staining of ruptured membranes. (**D**) An amniotic fluid macrophage (arrow head) attaches to ruptured amnion at 2 h (arrow). (**E** and magnified view in **F**) Amniotic fluid macrophages (green) attach to amnion surface at 24 h, and mesenchymal cells migrate (arrow head). Note mesenchymal-like shape change of epithelial cells in **F**. Bars, 50 µm. (**G** and **H**) Fluorescent *in situ* hybridization (FISH) of Y chromosome (red) and immunostaining for F4/80 (green) at healing amnion (**G**) and intact myometrium (**H**) at 48 h. Bars, 10 µm. (**I**–**L**) Macrophage adhesion molecules at the ruptured site. Immunostaining for F4/80 (green), VCAM1 (**I**) or P-selectin (**J**, red) and DAPI (blue) at ruptured amnion at 24 h after rupture by 20 G needle. Bars, 50 µm. (**K** and **L**) Gene expression of *VCAM1* (**K**) or *P-selectin* (**L**) of intact or ruptured fetal membrane with 20 G needle at indicated time. Error bars represent SEM. n = 5–6 fetal membranes from 5–6 pregnant mice in each group. *P < 0.05, **P < 0.01.

Innate immunity assists wound healing, and macrophages play a central role^10^. To investigate the involvement of macrophages in healing of the ruptured fetal membrane, immunostaining of macrophages was conducted at the healing site of both small (Fig. S4B,I) and large ruptured amnion 24 h after injury (Figs 6B and S4I). Two macrophage markers (F4/80 and CD68) were utilized indicating similar results regarding macrophage number and location. Macrophages were virtually absent in intact amnion before rupture (Figs 6A, S4A,I). In contrast, macrophages localized to the amnion 24 h after injury (Figs 6B and S4B) with rare macrophages in chorion and decidua (Fig. 6C). In contrast with other tissues in which neutrophils are recruited in the early phase of wound healing, neutrophils were rare in the healing amnion (Fig. S4C,D).

**Figure 7:**
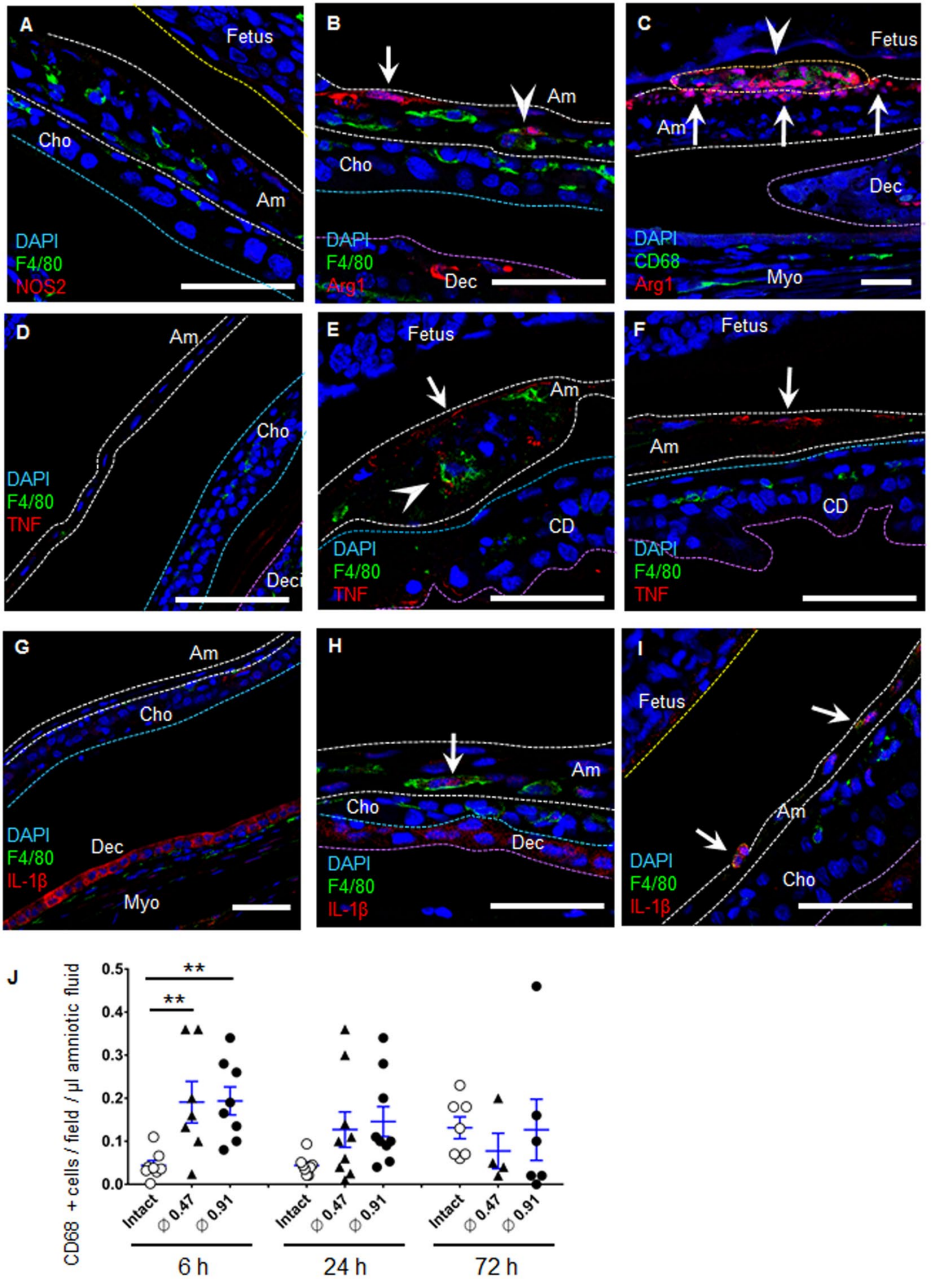
Localized cytokine secretion from M-2 macrophages. (**A**–**C**) Immunofluorescence staining of fetal membrane for F4/80 (green) or CD68 (green), NO synthase-2 (NOS2, red in **A**), arginase-1 (Arg1, red in **B**) and DAPI (blue) at 24 h of rupture by a 20 G needle (ø 0.91 mm). Dotted lines indicate amnion (white), surface of fetal skin (yellow), chorion (light blue), and decidua (purple). Note that Arg1 was expressed both in macrophages (**B** and **C**, arrowhead) and in amnion epithelial cells (**B** and **C**, arrow). Figure 7C is same location as Fig. 6E and F. Bars, 50 µm. (**D**–**I**) Immunofluorescence staining of fetal membrane for F4/80 (green), TNF (red in **D**–**F**), IL-1β (red in **G**–**I**) and DAPI (blue) by a 20 G needle (ø 0.91 mm). (**E** and **F**) TNF at healing site of amnion in macrophage (arrow head) and amnion epithelial cells (arrow) at 6 h after rupture. (**D**) Intact fetal membrane at 6 h. (**H** and **I**) IL-1β at healing amnion in macrophage (arrow) at 24 h after rupture. (**G**) Intact fetal membrane at 24 h. Bars, 50 µm. (**J**) Quantification of CD68-positive cells in the amniotic fluid of each gestational sac. Blue bar indicates mean and SEM. n = 4–9 from 2–5 pregnant mice in each group. **P < 0.01, ANOVA.

Next, we sought to determine the origin of macrophages recruited to the site of injury. Macrophages attached to the ruptured amnion as early as 2 h (Fig. 6D), and firmly attached to the ruptured site within 24 h (Figs 6E–F and 7C), with some incorporated within the amnion (Figs 6B,7 and S4). Remarkably, macrophage attachment was limited to the fetal side (Fig. 6D–F). In fetal membranes derived from male fetus, macrophages incorporated in the healing amnion were Y-chromosome positive using fluorescent *in situ* hybridization (FISH, Fig. 6G), confirming that these macrophages were of fetal origin. In contrast, macrophages in myometrium were Y-chromosome negative (maternal origin, Fig. 6H).

Adhesion molecules (e.g., intracellular adhesion molecule 1 (ICAM1), vascular cell-adhesion molecule 1 (VCAM1), E- and P-selectin) are expressed on the surface of inflamed sites in which macrophages attach^15^. Although immunostaining of VCAM1 and P-selectin was absent in intact amnion (Fig. S5B,E), both were localized to sites of amnion injury in both small (Fig. S5A,D) and large (Fig. 6I,J) ruptured sites. Macrophages adhered to P-selectin-expressing amnion within 24 h (Fig. 6J, arrow). Likewise, mRNA *VCAM1* and *P-selectin* increased as early as 2 h after both small and large ruptures (Figs 6K–L and S5C,F). Overall the data suggest that injured epithelial cells express adhesion molecules in response to injury likely to attach recruited amniotic macrophages for healing. Similar to the pattern of cytokine expression (Fig. 4), increased levels of *VCAM1* and *P-selectin* mRNA persisted after 20 G rupture (Fig. 6K,L), suggesting recruitment of macrophages continued until healing of amnion was complete. *E-selectin* mRNA also increased in the ruptured fetal membrane at 2 h but *ICAM1* gene expression was not regulated (data not shown).

Next, representative markers of M1-phenotype macrophages (NO synthase-2, NOS2) and M2 macrophages (arginase 1, Arg1) were used to characterize the phenotype of macrophages in the injured amnion^8^. In the healing site, these macrophages were NOS2-negative (Fig. 7A), even though myometrium stained positive with NOS2 (Fig. S4G). In contrast, Arg1-positive M2-macrophages were observed in the healing site of amnion (Fig. 7B,C). Thus macrophages at the ruptured amnion were M2 dominant, a wound healing phenotype, although the phenotype may be mixed in the early phases in which proinflammatory cytokines were localized to the injury. Unexpectedly, we also found strong staining of Arg1 in amnion epithelial cells (Fig. 7B,C).

In intact amnion, TNF and IL-1β were not observed (Fig. 7D,G). In ruptured site, TNF was expressed in macrophages and amnion epithelial cells (Fig. 7E). Similarly, IL-1β was expressed in macrophages (Figs 7H,I and S7F). IL-1β was also expressed in intact decidua (Fig. 7G). Remarkably, secretion of these cytokines was localized to the ruptured site of amnion. Interestingly, the number of CD68-positive macrophages per volume of amniotic fluid was increased significantly by ruptured membranes within 6 h (Fig. 7J). At 24–72 h, increased density of macrophages in amniotic fluid tended to return to basal level, suggesting that amniotic fluid floating-macrophages attach and are incorporated into the wounded amnion within 24 hours. Together, cytokine release is highly localized at the healing site of amnion by macrophages and responsive epithelial cells.

### Epithelial-mesenchymal transition (EMT) by IL-1β and TNF

**Figure 8:**
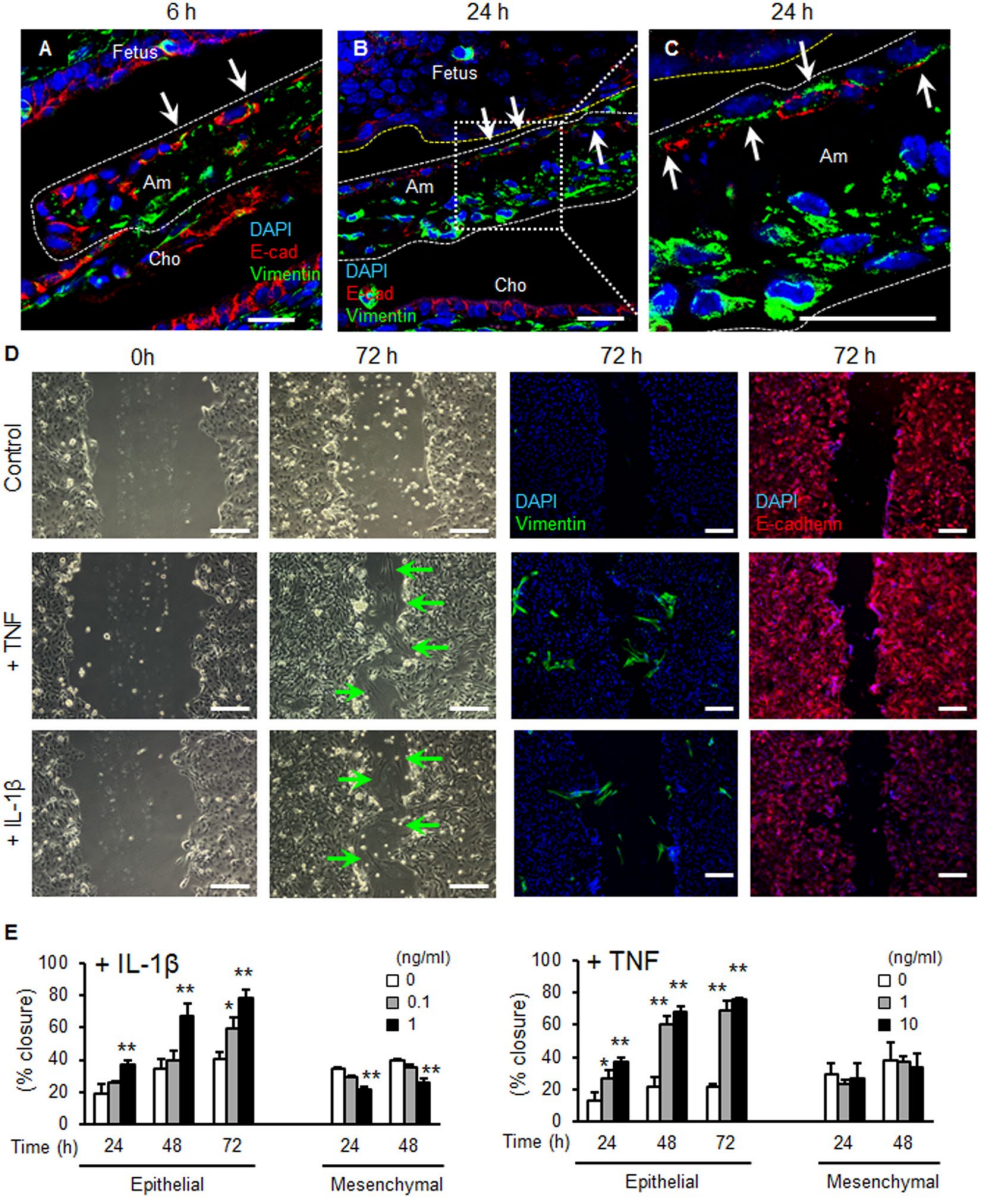
Epithelial-mesenchymal transition (EMT) in mouse pPROM and human amnion epithelial cells. (**A**–**C**) Immunostaining for vimentin (green), E-cadherin (red) and DAPI (blue) at ruptured site of amnion by a 20 G needle (ø 0.91 mm). Ruptured amnion after 6 h (**A**, 26 G) and 24 h (**B** and **C**, 20 G**)**. (**C**) is a magnified image of (**B**). Note that vimentin-positive cells were observed in the epithelial layer of amnion (arrow). Bars, 20 µm. (**D** and **E**) Wound healing scratch assay of primary human amnion epithelial cells. Cells were treated with vehicle control, IL-1β (1 ng/ml) or TNF (10 ng/ml) after scratching. Phase contrast or immunostaining images with vimentin (green), E-cadherin (red), or DAPI (blue) were obtained at 0 or 72 h. Green arrow indicates mesenchymal-like shape cells in wounded area of epithelial cells. Bars, 200 µm. (**E**) Percent closure of scratched human amnion epithelial and mesenchymal cells treated with different doses of IL-1β (upper panel) or TNF (lower panel). Error bars represent SD. n = 3 in each group. *P < 0.05, **P < 0.01, compared to control (0 ng/ml) at each time point.

Epithelial-mesenchymal transition (EMT) is a biological process that changes epithelial cell phenotypes to a mesenchymal cell phenotype. Since EMT greatly enhances cellular migration and tissue repair, we investigated the possibility that EMT may participate in healing of the injured amnion. Vimentin-positive or E-cadherin-vimentin double-positive cells were scattered in the epithelial cell layer of the injured amnion within 6 h (Figs 8A and S6F). These vimentin-positive cells were also observed at 24 h (Figs 8B,C and S6F). The findings that epithelial layers are clearly separated from accumulation of the mesenchymal cell layer in the healing site of amnion suggests the possibility of EMT *in vivo*.

Proinflammatory cytokines have potential to induce EMT in healing of skin^16^. Thus, we tested whether IL-1β or TNF induce EMT in primary human amnion epithelial cells. In wound healing scratch assays of amnion epithelial cells, migration was increased dose-dependently by IL-1β or TNF (Fig. 8D,E). In contrast, IL-1β or TNF did not stimulate migration of mesenchymal cells (Fig. 8E). Closure of scratch is considered to be stimulated by migration, not proliferation, of epithelial cells, because IL-1β or TNF did not change the number of viable cells in proliferation assay of amnion epithelial cells (data not shown). In addition, IL-6 did not stimulate migration of either epithelial or mesenchymal cells of amnion (data not shown). In the scratched area, IL-1β or TNF changed the morphology of amnion epithelial cells into spindle-shaped cells (Fig. 8D, green arrows in second column). Immunofluorescence revealed that these spindle-shaped cells were vimentin-positive (Fig. 8D, 3rd column), suggesting EMT, and vimentin-positive cells were also scattered in the non-wounded area (Fig. 8D, 3rd column). Vimentin-positive cells were not observed in control cells without injury. IL-1β and TNF also changed morphology of epithelial cells to spindle-shaped (Fig. 8D, 2nd column). Most of these cells maintained an epithelial phenotype (i.e., E-cadherin positive and vimentin-negative) (Fig. 8D, 3rd and 4th columns) despite the change in epithelial cell shape. Extension of culture for 5 days resulted in appearance of vimentin-positive cells in the scratched area of primary human amnion epithelial cells even without cytokine treatment (Fig. S6C). Gene expression demonstrated that *vimentin* mRNA was significantly increased in scratched cells compared to control cells without injury, whereas *E-cadherin* mRNA was not changed (Fig. S6D). Moreover, culture with 10% FBS for 8 days dramatically increased EMT of amnion epithelial cells (Fig. S6A,B). Since FBS includes various cytokines and growth factors, we suggest that amnion epithelial cells readily undergo EMT after stimulation with these factors. The rate of epithelial cell migration is slower than that of mesenchymal cells (Fig. S5E). EMT would thereby increase the migration rate of epithelial cells thereby accelerating closure of the ruptured amnion. Some of the massive mesenchymal cells aggregating in the healing center *in vivo* may be derived from cytokine-stimulated amnion epithelial cells through EMT.

## Discussion

Here, we investigated the mechanisms of healing of sterile preterm premature rupture of the fetal membranes. Using a preclinical model of pPROM in mice and primary human amnion cells in culture, we demonstrated that (i) sterile small rupture of amnion at embryonic day 15 healed spontaneously,(ii) healing of sterile large rupture of amnion was incomplete within 72 h, (iii) Arg1-positive amniotic fluid fetal macrophages were recruited to the site of injury and incorporated into the amnion at later time points, and (iv) proinflammatory cytokines, IL-1β and TNF, secreted from macrophages recruited at early time points, stimulated EMT of amnion epithelial cells.

### Healing of fetal membranes

In our sterile puncture model, we found that small ruptures of fetal membranes in mid-gestation (ø 0.47 mm) heal within 3 days. Perhaps, this is not surprising in that small puncture of fetal membranes by amniocentesis in humans heals spontaneously^17^. Nonetheless, healing of 0.47 mm punctures in mice illustrates that (i) the amnion exhibits striking regenerative potential^18^, and (ii) embryonic wound healing is rapid and complete compared with adult tissues^7,19^. In addition, the surface of migrating amnion mesenchymal cells was covered by a monolayer of epithelial cells. These morphological features were similar to the classical report of sterile rupture of rat fetal membranes in a thickened edge of amnion appeared as a bulge with a pointed tip at 24 h and fibrous spindle-shaped mesenchymal cells were covered by a single layer of flattened epithelial cells^20^. Nevertheless, healing rates of large rupture size (ø 0.91 mm) were only 40%. This is compatible with clinical findings in which comparably large rupture sizes caused by fetoscopy do not heal^21^. Although choriodecidua does not contribute to the load-bearing capacity of the fetal membranes and not the focus of this investigation, healing of choriodecidua was slow and incomplete compared with amnion. We speculate that decidual tissue of maternal origin exhibits slow rates of healing relative to fetal cells of the amnion. Further, macrophages readily observed after injury in the amnion were rare after injury of the decidua, suggesting that choriodecidua is less assisted by innate immunity of wound-healing.

Interestingly, amniotic fluid volume did not recovered despite closure of the amnion. During normal gestation in the mouse, amniotic fluid decreases significantly during late gestation (from ~120 to 55 µl, Fig. 3C) suggesting that failure to increase amniotic fluid after rupture of the membranes on d15 may simply represent the normal negative balance of fluid accumulation at this time in gestation. This result is also supported by the previous report that amniotic fluid volume decreased significantly in mice from ED 15 to ED 18, unlike human^22^, indicating that absorption of amniotic fluid overcomes the synthesis toward term. In addition, although we histologically confirmed closure of amnion, closure of choriodecidua was not complete at 72 h (Fig. S2). It is possible that lack of full fetal membrane repair resulted in continuous leakage of amniotic fluid. One of the risks of prolonged rupture of the membranes is lack of fetal lung maturity. Examination of fetal lung 3 d after rupture, however, did not reveal negative effects of the modest oligohydramnios during this time in gestation.

Various forms of biological materials have been used to repair preterm premature ruptured membranes including fibrin glue, platelet rich plasma, cryoprecipitates, fibrinogen, thrombin, polytetrafluoroethylene material, matrigel, and gelatin sponges^23^. However the results of these trials were not necessarily satisfactory or remain at the preclinical level. We believe that our mouse pPROM model will be useful for investigating basic mechanisms and treatment of pPROM.

### Macrophage infiltration

The presence of macrophages early after injury of the amnion was interesting since resident macrophages were not observed in intact amnion and the avascular nature of the amnion would preclude macrophages derived from circulating monocytes. Three potential sources of amnion macrophages were considered: 1) decidua, 2) amniotic fluid, or 3) differentiation of resident amnion stem cells. Appearance of macrophages at the site of injury as early as 2 h of rupture speaks against the possibility of monocyte differentiation which requires several days^24^. Likewise, differentiation of resident stem cells is unlikely in this time frame. Recruitment of decidual macrophages into amnion was considered because decidua is enriched in resident macrophages^13^ However, macrophages were not observed at the wounded choriodecidua. Further, wound-induced accumulation of amnion macrophages were attached to the fetal side of amnion (epithelium) where adhesion molecules P-selectin and VCAM1 were highly expressed but not the adjacent mesenchymal cell layer. Further, these cells were Y positive in the case of a male fetus. Collectively, these results indicate that amniotic fluid is the origin of amnion-healing macrophages. The concept of “migrating amniotic fluid macrophage” is also reported in other situations during pregnancy^25,26^. Physiologically, it is known that the number of amniotic fluid macrophages increases toward term in mouse^27^. In our data, consistent with the previous report, the number of macrophages in amniotic fluid increased 6 h (ED 15) to 72 h (ED18) (Fig. 7J). These increased amniotic fluid macrophages are considered to infiltrate myometrium and activate myocytes to initiate parturition^25,26^.

In addition to macrophages, Arg1 was also expressed in amnion epithelial cells. This phenomenon in which Arg1 is highly expressed in inflamed epithelial cells has been reported in other tissues (e.g., lung epithelial cells of patients with asthma^28^ and skin epidermis of patients with psoriasis^29^). Since inhibition of Arg1 activity delays cutaneous healing^30^, we pose that arginase-1 is not only a marker of M2 macrophages but also plays a role in healing of the amnion.

In the process of wound healing, macrophages phagocytose organisms and matrix debris, and release various growth factors and cytokines. In this study, we observed secretion of IL-1β and TNF from macrophages which stimulated EMT of amnion epithelial cells. EMT is observed during embryogenesis, organ development, wound healing, tissue regeneration, and cancer and its metastasis^31^. Cell-cell contacts are important in the maintenance of an epithelial phenotype. Disruption of cell-cell contacts initiates EMT via cadherin switching^32^, which resulted in generation of mesenchymal-shaped cells in our mouse model and primary amnion cells. Thus, although macrophages may serve a positive role in healing acutely, excessive or chronic inflammation delays wound healing^33^. In our model of sterile pPROM, increased expression of the anti-inflammatory cytokine IL-10 accompanied amplified expression of inflammatory cytokines. IL-10 inhibits production of proinflammatory cytokines (e.g., IL-1α, IL-1β, TNF, and IL-6) from activated macrophage^34^. Further, treatment of cutaneous wounds with IL-10 neutralizing antibodies results in overexpression of chemokines and cytokines and exaggerated production of neutrophil chemoattractants^35^. In this ruptured membrane model, secretion of IL-1β and TNF was well-localized and limited, suggesting that anti-inflammatory mechanisms (e.g., IL-10) tightly regulate the inflammatory response to optimize wound healing of amnion. It should also be emphasized that these unique mechanisms become crucial in a tissue that cannot depend on growth factors and other stimulants from the vasculature.

### Overall mechanisms of membrane healing: small vs large injury

Our mouse models of pPROM indicate that small ruptures heal but the majority of large ruptures do not. Comparisons between the two models reveal potential mechanisms for these differences. First, cytokine and chemokine responses are transient after small rupture, but prolonged after larger injury. Second, *MMP9* gene expression is upregulated dramatically in the later stages of wound healing in the large rupture but not small. Third, residual amniotic fluid is present after small, but not large, rupture. This amniotic fluid, although decreased relative to intact membranes, may serve as a reservoir for macrophages and other healing factors or their binding proteins. Regardless, the major problem in healing after large rupture seems to be the lack of mesenchymal cell migration and matrix deposition. In the case of small ruptures, EMT may be sufficient to bridge matrix support and strength of the membrane. After large ruptures, however, it is likely to be insufficient to scaffold the large mesenchymal defect, especially in the presence of protease activation.

### Summary

In conclusion, we analyzed in detail spontaneous healing of the fetal amnion using preclinical models of small and large premature rupture of the membranes. We conclude that amnion has high regenerative potential through unique mechanisms including (i) recruitment of fetal macrophages from the amniotic fluid, and (ii) IL-1β- and TNF-induced EMT and migration of amnion epithelial cells. Healing of large ruptures is compromised likely due to defects in mesenchymal cell migration and matrix deposition. We suggest, therefore, that the model is an interesting first step in understanding the role of “sterile inflammation” and healing of the amnion.

## Materials and Methods

### Preclinical model of sterile ruptured membrane

All animals were handled and euthanized in accordance with the standards of humane animal care described by the National Institutes of Health Guide for the Care and Use of Laboratory Animals, using protocols approved by the Institutional Animal Care and Use Committee (IACUC) of the University of Texas Southwestern Medical Center at Dallas. On 15 days post coitum (dpc), C57/Bl6 pregnant mice were anesthetized with isoflurane. A ventral incision was made and uterus and fetal membranes punctured with sterile needles (26 or 20 gauge). In each uterus, half of gestational sacs were punctured (rupture group) and half were not (intact control). In the case of 20 G injury, 4 punctures were conducted per sac whereas 8 punctures per sac were conducted with 26 G needles. Rupture of membrane was confirmed by leakage of amniotic fluid. To mark puncture sites, needles were soaked in 10% India Ink before puncture resulting in black staining of the puncture site with contact of the outer surface of the needle. After each procedure, the maternal abdomen was closed with 5-0 Polysorb. At the time of sampling, diameter of rupture was measured by a digital caliper under microscopy. Closure was defined as an invisible hole under microscopy and the membrane continuous at the rupture site. Fetal membranes were collected after washing with PBS, quick frozen in liquid nitrogen, and stored at −80 °C. All procedures adhered to the NIH Guide for the Care and Use of Laboratory.

### Scanning Electron Microscope (SEM)

Fetus, fetal membranes, and placenta were fixed with 2.5% (v/v) glutaraldehyde in 0.1 M sodium cacodylate buffer overnight at 4 °C. After three rinses in 0.1 M sodium cacodylate buffer, they were post-fixed with 2% osmium tetroxide in the same buffer for 2 h. Samples were rinsed with water and dehydrated with increasing concentration of ethanol, followed by critical point drying (Tousimis Samdri-795). Dried fetal membrane was cut around the placenta, and fetus and placenta were removed. Fetal membranes were mounted on SEM stubs like a “shell” and fixed with the inside of the fetal membrane shell (amnion side) upward. Membranes were sputter coated with gold/palladium (Cressington 108 auto sputter coater) and images acquired on a Field-Emission Scanning Electron Microscope (Zeiss Sigma) at 3.0 kV accelerating voltage.

### Quantitative real-time PCR

Non-injured tissue around the rupture site would dilute the changes of mRNA level, so we made multiple punctures per gestation sac to increase the ruptured area. In addition, we cut off non-ruptured site of fetal membrane as far as possible at sampling. Quantitative RT-PCR was used to determine the relative levels of gene expression. Primer sequences for amplifications are shown in Supplementary Table 1. SYBR green to detect amplification and gene expression was normalized to that of indicated housekeeping gene.

### Isolation and culture of amnion epithelial and mesenchymal cells

Separation and isolation of human amnion epithelial and mesenchymal cells were performed as previously described^36^. The protocol for obtaining placentae and fetal membranes was approved by the Institutional Review Board of the University of Texas Southwestern Medical Center. Briefly, amnion tissue was separated by blunt dissection and then minced. Cells were dispersed by enzymatic digestion with trypsin (Trypsin 1:250, #27250-018, Gibco), and then collagenase (Collagenase B, #11088831001, Roche) and DNase I (10104159001, Roche). Isolated amnion cells were suspended in DMEM/F12 (D8437, Sigma) with 10% fetal bovine serum and 1% antibiotic-antimycotic solution (Anti Anti 100X, #15240-062, Gibco). Cells were plated in plastic culture dishes, maintained at 37 °C in a humidified atmosphere of 5% CO_2_ in air, and allowed to replicate in monolayer to confluence.

### Immunofluorescent staining

Mouse uterus was fixed in 4% paraformaldehyde overnight and kept in 50% ethanol until paraffin embedding. To ensure that sites of injury were localized, sections were targeted at the black stain left by India Ink needles. Second, serial sections were taken every 500 µm throughout the injury, and serial sections of nonruptured sacs were also obtained to ensure that the defects were not due to preparation artefacts. After sections (5 μm) were deparaffinized, antigen retrieval was performed by incubation with proteinase K (P8107S, New England Biolab, working concentration, 0.6 units/ml) for 1 minute at 37 °C. For antigen retrieval of CD68, E-cadherin, and vimentin, sections were boiled in 10 mM sodium citrate buffer (pH 6.0) by microwave for 20 min, and then cooled for 30 min at room temperature. After antigen retrieval, sections were preincubated with 10% normal goat serum (50062Z, Life Technologies) with 0.3% Triton X-100 for 30 min at room temperature. Subsequently, tissue sections were incubated with primary antibody in PBS with 1% BSA and 0.3% Triton X-100 at 4 °C overnight. Primary antibodies used and concentration were as follows: F4/80 (clone BM8, eBioscience, #14-4801, 1:50), Vimentin (D21H3, Cell Signaling, #5741, 1:100), E-cadherin (4A2, Cell Signaling, #14472, 1:50), VCAM1 (EPR5047, Abcam, #134047, 1:100), P-selectin (CD62P, Abcam, #202983, 1:100), NOS2 (iNOS, Abcam, #15323, 1:100), Arg1 (Arginase-1, D4E3M, Cell Signaling, #93668, 1:100), TNF (Abcam, #6671, 1:100), IL-1β (Abcam, #9722, 1:100), Ly-6G (eBioscience, #14-5931, 1:100), CD68 (KP1, Abcam, #955, 1:100), Ki67 (Abcam #15580, 1:100), HSP70 (Abcam #2787, 1:100). Thereafter, sections were incubated with Alexa Fluor 488, 549, or 594–conjugated secondary antibodies (Ig, heavy and light chains, Invitrogen, 1:500) in 10% normal goat serum for 1 h at room temperature. After that, slides were mounted with Prolong Gold Antifade Reagent with DAPI (P36935, Molecular Probes). Images were taken by Leica SP5 confocal microscopy. ImageJ software was used to generate individual images.

### Fluorescence *in situ* hybridization of Y chromosome

Images of sections immunostained with F4/80 and DAPI (antigen retrieval, proteinase K at 37 °C for 1 minute) were obtained before hybridization using Leica SP5 confocal microscopy. Thereafter, the cover glass was removed by shaking in warmed PBS (37 °C, 30 min)., sections were washed by 2X saline-sodium citrate buffer (SSC), and then pretreated with 10 mM sodium citrate (pH 6.0)/2 mM EDTA (pH 8.0) at 80 °C for 45 min. After washing with 2X SSC, sections were dehydrated with 70% ethanol (1 minute) then 100% ethanol (1 minute). Dry sections and murine Y chromosome probe (Cytocell, AMP0YR) were pre-warmed at 37 °C for 5 min. 10 µl of FISH probe was applied for 22 mm × 22 mm area, and covered with cover glass and sealed with rubber cement. After glue was dry, sections were denatured on hot-plate at 75 °C for 5 min. Sections were then incubated in humidified box at 37 °C overnight. After removing cover glass, sections were washed with 2X SSC (room temperature, 5 min), 2X SSC/0.3% NP-40 (75 °C, 5 min), then 2X SSC (room temperature, 1 min), and mounted with Prolong Gold Antifade Reagent with DAPI. Images were obtained with Leica SP5 confocal microscopy. The fields of immunostaining and FISH images were matched according to the location of nuclear distribution to be as close to each other as possible^37^. ImageJ software was used for image processing.

### Wound healing scratch assay

Primary human amnion epithelial or mesenchymal cells were harvested in 12-well plate. At confluency, the center of each well was scratched with a 10 µl tip (epithelial cells) or a 200 µl tip (mesenchymal cells). After washing with PBS, IL-1β (#201-LB, R&D Systems), or TNF (#210-TA, R&D Systems) was added in serum-free medium. The width of wound was measured microscopically with 3 points in each field. Three different fields were selected in each well, and width was averaged. Percent closure was calculated by dividing the average width of the indicated time by the width of 0 h.

### Immunocytochemistry

Primary human amnion epithelial or mesenchymal cells were grown in 8-well chamber slide. Cells fixed in 4% paraformaldehyde for 10 min. After washing with PBS, slides were incubated with 10% normal goat serum for 30 min at room temperature. Thereafter, slides were incubated with primary antibodies overnight at 4 °C. Primary antibodies used and concentration was as follows: Vimentin (D21H3, Cell Signaling, #5741, 1:100), E-cadherin (4A2, Cell Signaling, #14472, 1:50), and CD68 (KP1, Abcam, #955, 1:100). After incubation with secondary antibody (Alexa Fluor 488, 549, or 594, Invitrogen, 1:500 dilution), slides were mounted with Prolong Gold DAPI. Images were taken by Leica SP5 confocal microscopy, and generated by ImageJ software.

### Isolation of mouse amniotic fluid macrophage

Amniotic fluid macrophages were isolated from mice as described previously^26^. Briefly, amniotic fluid from individual sacs was aspirated with a 20-gauge needle with 1.0-ml syringe containing 100 µl of RPMI medium 1640. Collected amniotic fluid of each gestational sac was added to the 8-well chamber slide, and incubated at 37 °C for 1 h in incubator. After confirming cell attachment to the slide, cells were fixed with 4% paraformaldehyde for 10 min, and then processed for immunocytochemistry. For quantification of macrophages, five fields were randomly selected using 20× objective and the average number of CD68-positive cells per field was divided by the amniotic fluid volume (µl).

### Statistical Analysis

Values were expressed as means ± SD *in vitro* experiments or means ± SEM in mice experiments. Data were analyzed by unpaired Student’s *t*- test unless indicated otherwise. For comparison of diameter of rupture of amnion and choriodecidua, and number of CD68 positive cells in amniotic fluid, ANOVA was conducted. For complete closure of amnion, chi-square test was utilized. *P*-values less than 0.05 were regarded as statistically significant.

## Electronic supplementary material

Supplementary Information

## References

[1] Parry S, Strauss JF (1998). Premature rupture of the fetal membranes. N Engl J Med.

[2] Goldenberg RL, Culhane JF, Iams JD, Romero R (2008). Epidemiology and causes of preterm birth. Lancet.

[3] Romero R (1988). Intraamniotic infection and the onset of labor in preterm premature rupture of the membranes. Am J Obstet Gynecol.

[4] Gomez R (1998). The fetal inflammatory response syndrome. Am J Obstet Gynecol.

[5] Johnson, J. W., Egerman, R. S. & Moorhead, J. Cases with ruptured membranes that “reseal”. *Am J Obstet Gynecol* **163**, 1024–1030; discussion 1030–1022 (1990). 10.1016/0002-9378(90)91117-u 2206055

[6] Martin P (1997). Wound healing–aiming for perfect skin regeneration. Science.

[7] Sonnemann KJ, Bement WM (2011). Wound repair: toward understanding and integration of single-cell and multicellular wound responses. Annu Rev Cell Dev Biol.

[8] Gordon S (2003). Alternative activation of macrophages. Nat Rev Immunol.

[9] Gordon S, Martinez FO (2010). Alternative activation of macrophages: mechanism and functions. Immunity.

[10] Murray PJ, Wynn TA (2011). Protective and pathogenic functions of macrophage subsets. Nat Rev Immunol.

[11] Underwood MA, Gilbert WM, Sherman MP (2005). Amniotic fluid: not just fetal urine anymore. J Perinatol.

[12] Sutherland GR, Bauld R, Bain AD (1974). Observations on human amniotic fluid cell strains in serial culture. J Med Genet.

[13] Sutton L, Mason DY, Redman CW (1983). HLA-DR positive cells in the human placenta. Immunology.

[14] Shaw TJ, Martin P (2009). Wound repair at a glance. J Cell Sci.

[15] Imhof BA, Aurrand-Lions M (2004). Adhesion mechanisms regulating the migration of monocytes. Nat Rev Immunol.

[16] Yan C (2010). Epithelial to mesenchymal transition in human skin wound healing is induced by tumor necrosis factor-alpha through bone morphogenic protein-2. Am J Pathol.

[17] Borgida AF, Mills AA, Feldman DM, Rodis JF, Egan JF (2000). Outcome of pregnancies complicated by ruptured membranes after genetic amniocentesis. Am J Obstet Gynecol.

[18] Maxson S, Lopez EA, Yoo D, Danilkovitch-Miagkova A, Leroux MA (2012). Concise review: role of mesenchymal stem cells in wound repair. Stem Cells Transl Med.

[19] Redd MJ, Cooper L, Wood W, Stramer B, Martin P (2004). Wound healing and inflammation: embryos reveal the way to perfect repair. Philos Trans R Soc Lond B Biol Sci.

[20] Sopher D (1972). The response of rat fetal membranes to injury. Ann R Coll Surg Engl.

[21] Gratacos E (2006). A histological study of fetoscopic membrane defects to document membrane healing. Placenta.

[22] Cheung CY, Brace RA (2005). Amniotic fluid volume and composition in mouse pregnancy. Journal of the Society for Gynecologic Investigation.

[23] Devlieger R, Millar LK, Bryant-Greenwood G, Lewi L, Deprest JA (2006). Fetal membrane healing after spontaneous and iatrogenic membrane rupture: a review of current evidence. Am J Obstet Gynecol.

[24] Geissmann F (2010). Development of monocytes, macrophages, and dendritic cells. Science.

[25] Thomson AJ (1999). Leukocytes infiltrate the myometrium during human parturition: further evidence that labour is an inflammatory process. Hum Reprod.

[26] Condon JC, Jeyasuria P, Faust JM, Mendelson CR (2004). Surfactant protein secreted by the maturing mouse fetal lung acts as a hormone that signals the initiation of parturition. Proc Natl Acad Sci USA.

[27] Kobayashi K, Umezawa K, Yasui M (2011). Apoptosis in mouse amniotic epithelium is induced by activated macrophages through the TNF receptor type 1/TNF pathway. Biol Reprod.

[28] North ML, Khanna N, Marsden PA, Grasemann H, Scott JA (2009). Functionally important role for arginase 1 in the airway hyperresponsiveness of asthma. Am J Physiol Lung Cell Mol Physiol.

[29] Bruch-Gerharz D (2003). Arginase 1 overexpression in psoriasis: limitation of inducible nitric oxide synthase activity as a molecular mechanism for keratinocyte hyperproliferation. Am J Pathol.

[30] Campbell L, Saville CR, Murray PJ, Cruickshank SM, Hardman MJ (2013). Local arginase 1 activity is required for cutaneous wound healing. J Invest Dermatol.

[31] Kalluri R, Weinberg RA (2009). The basics of epithelial-mesenchymal transition. J Clin Invest.

[32] Wheelock MJ, Shintani Y, Maeda M, Fukumoto Y, Johnson KR (2008). Cadherin switching. J Cell Sci.

[33] Eming SA, Martin P, Tomic-Canic M (2014). Wound repair and regeneration: mechanisms, signaling, and translation. Sci Transl Med.

[34] Moore KW, de W Malefyt R, Coffman RL, O’Garra A (2001). Interleukin-10 and the interleukin-10 receptor. Annu Rev Immunol.

[35] Sato Y, Ohshima T, Kondo T (1999). Regulatory role of endogenous interleukin-10 in cutaneous inflammatory response of murine wound healing. Biochem Biophys Res Commun.

[36] Casey ML, MacDonald PC (1996). Interstitial collagen synthesis and processing in human amnion: a property of the mesenchymal cells. Biol Reprod.

[37] Miyata E (2008). Hematopoietic origin of hepatic stellate cells in the adult liver. Blood.

