# Supplementary material for: Healing of Preterm Ruptured Fetal Membranes
Source: Sci Rep. 2017 Oct 13;7:13139. doi: 10.1038/s41598-017-13296-1 (PMC5640674; doi:10.1038/s41598-017-13296-1)
Supplement: Supplementary file 1 — Supplementary Information [file 41598_2017_13296_MOESM1_ESM.pdf]

Healing of Preterm Ruptured Fetal Membranes

Haruta Mogami[1], Annavarapu Hari Kishore[1], Yucel Akgul[2] and R. Ann Word[1]

[1]The Cecil H. and Ida Green Center for Reproductive Biological Sciences
Department of Obstetrics and Gynecology
University of Texas Southwestern Medical Center
Dallas, Texas

[2]Department of Plastic Surgery
University of Texas Southwestern Medical Center
Dallas, Texas

Supplementary Table 1. Primer sequences used for qPCR

Mouse

Gene	Forward	Reverse
bFGF	5'- CCACACGTCAAACTACAACTCCAA -3'	5'- TTGGCACACACTCCCTTGATAG -3'
β2 microglobulin	5'- CCGAGCCCAAGACCGTCTA -3'	5'- AACTGGATTTGTAATTAAGCAGGTTCA -3'
COL1A1	5'- GCCTTGGAGGAAACTTTGCTT -3'	5'- GCACGGAAACTCCAGCTGAT -3'
COL3A1	5'- CACAGCAGTCCAACGTAGATGAA -3'	5'- TGACATGGTTCTGGCTTCCA -3'
COL5A1	5'- CTCGACTGTAAGAAGAAGATTACGAAGT -3'	5'- CTCAAATATTTCATCATCCAGAATCC -3'
EGF	5'- ACGTAGGTCACCCCTTCTCTCTAG -3'	5'- CCGATGGGATAGCCCAATC -3'
E-selectin	5'- AAAGCAACTGCTGGAGTCATGA -3'	5'- TCCAGCGAGGAGAACAAAAAC -3'
FGF1	5'- CCAGCCTGCCAGTTCTTCAG -3'	5'- GGCTGCGAAGGTTGTGATCT -3'
ICAM1	5'- GCTGTTTGAGCTGAGCGAGAT -3'	5'- AACGAATACGCGGTGATGGT -3'
IGF1	5'- CTTCAACAAGCCCACAGGCTAT -3'	5'- AAGCAACACTCATCCACAATGC -3'
IGF2	5'- CCGTACTTCCGGACGACTTC -3'	5'- CGTCCCGCGGACTGTCT -3'
IL10	5'- GGACCAGCTGGACAACATACTG -3'	5'- GCAACCCAAGTAACCCTTAAAGTC -3'
IL1β	AAGATGAAGGGCTGCTTCCA -3'	5'- TGAGTGATACTGCCTGCCTGAA -3'
MMP2	GGACTATGACCGGGATAAGAAATATG -3'	5'- GGGCACCTTCTGAATTTCCA -3'
MMP9	AGACCAAGGGTACAGCCTGTTC -3'	5'- GCACGCTGGAATGATCTAAGC -3'
PDGFA	CTCGAAGTCAGATCCACAGCAT -3'	5'- CAGCCCCTACGGAGTCTATCTC -3'
P-selectin	CGTCTCAGAAAGAAAGATGATGGA -3'	5'- GCGTTAGTGAAGACTCCGTATGTTC -3'
TGFβ1	AGCGCTCACTGCTCTTGTGA -3'	5'- GCTGATCCCGTTGATTTCCA -3'
TGFβ2	CCTTCGTGCCGTCTAATAATTACA -3'	5'- TGCCATCAATACCTGCAAATCT -3'
TGFβ3	GGCAGAGTTCCGGGTCTTG -3'	5'- CGAAGTATCTGGAAGAGCTCAATTC -3'
TNF	GACCCTCACACTCAGATCATCTTCT -3'	5'- TCCTCCACTTGGTGGTTTGC -3'
VCAM1	CTACAAGTCTACATCTCTCCCAGGAAT -3'	5'- CACAGCACCACCCTCTTGAA -3'
VEGF	AGCAGAAGTCCCATGAAGTGATC -3'	5'- TCAATCGGACGGCAGTAGCT -3'

Human

β2 microglobulin	5'- CGCTCCGTGGCCTTAGC -3'	5'- AATCTTTGGAGTACGCTGGATAGC -3'
Cyclophilin A	5'- CCACCGTGTTCTTCGACATTG -3'	5'- TTTCTGCTGTCTTTGGGACCTT -3'
GAPDH	5'- GGAGTCAACGGATTTGGTCGTA -3'	5'- CAACAATATCCACTTTACCAGAGTTA -3'
E-cadherin	5'- TGGGCAGAGTGAATTTTGAAGA -3'	5'- TGAAACCGTAGAGGCCTTTTGA -3'
Vimentin	5'- GGTTGATACCCACTCAAAAAGGA -3'	5'- TTCGTTGATAACCTGTCCATCTCTAG -3'

Supplementary Table 2, mRNA levels of alveolar epithelial markers.

Gene expression of fetal lung (72 h after rupture, 18.5 d.p.c.) was analyzed by qPCR. Relative mRNA expression (normalized to that of β2 microglobulin) of each gene was compared with levels in intact membrane. Values are mean ± SEM. n=3-7 in each group.

	**Intact**	**ø 0.47mm**	**ø 0.91mm**
Amniotic fluid volume (μl)	64.3 ± 8.7	16.3 ± 5.5*	15.8 ± 3.7*
Alveolar type 1 cells	Relative gene expression		
PDPN	1.01 ± 0.07	1.01 ± 0.07	1.00 ± 0.04
Alveolar type 2 cells			
SFTPA1	1.01 ± 0.06	1.01 ± 0.01	1.15 ± 0.05
SFTPB	1.01 ± 0.04	1.18 ± 0.05	1.27 ± 0.06
SFTPC	1.02 ± 0.09	1.26 ± 0.20	1.35 ± 0.12
STFPD	1.01 ± 0.06	0.93 ± 0.02	1.08 ± 0.06
MUC1	1.01 ± 0.06	1.14 ± 0.05	1.23 ± 0.05
Nkx2.1	1.02 ± 0.08	1.14 ± 0.07	1.17 ± 0.06
ABCA3	1.01 ± 0.05	1.13 ± 0.04	1.14 ± 0.07

*P < 0.01 compared with intact

PDPN: Podoplanin, *SFTPA1*: surfactant protein A1, *SFTPB*: surfactant protein B, *SFTPC*: surfactant protein C, *SFTPD*: surfactant protein D, *MUC1*: Mucin 1, *Nkx2.1*: NK2 homeobox 1, *ABCA3*: ATP-binding cassette sub-family A member 3.

Figure S1

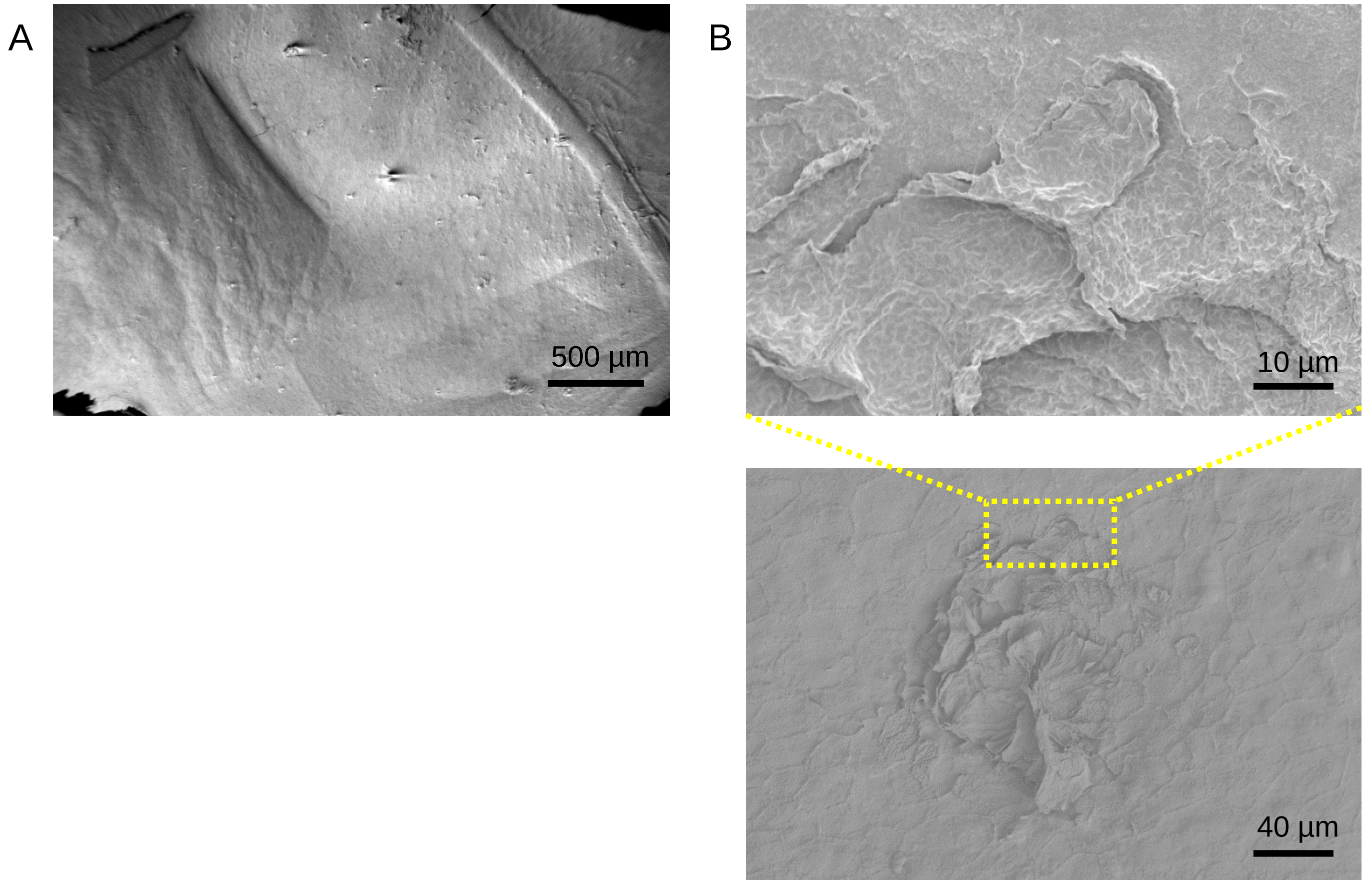

Figure S1. Images of scanning electron microscopy (SEM). **(A)** Intact non-ruptured amnion from inside the gestational sac at 72 h (embryonic day 18.5). **(B)** Healed amnion 72 h after injury (higher resolution). **(C)** Magnified image of Figure 2C.

Figure S2

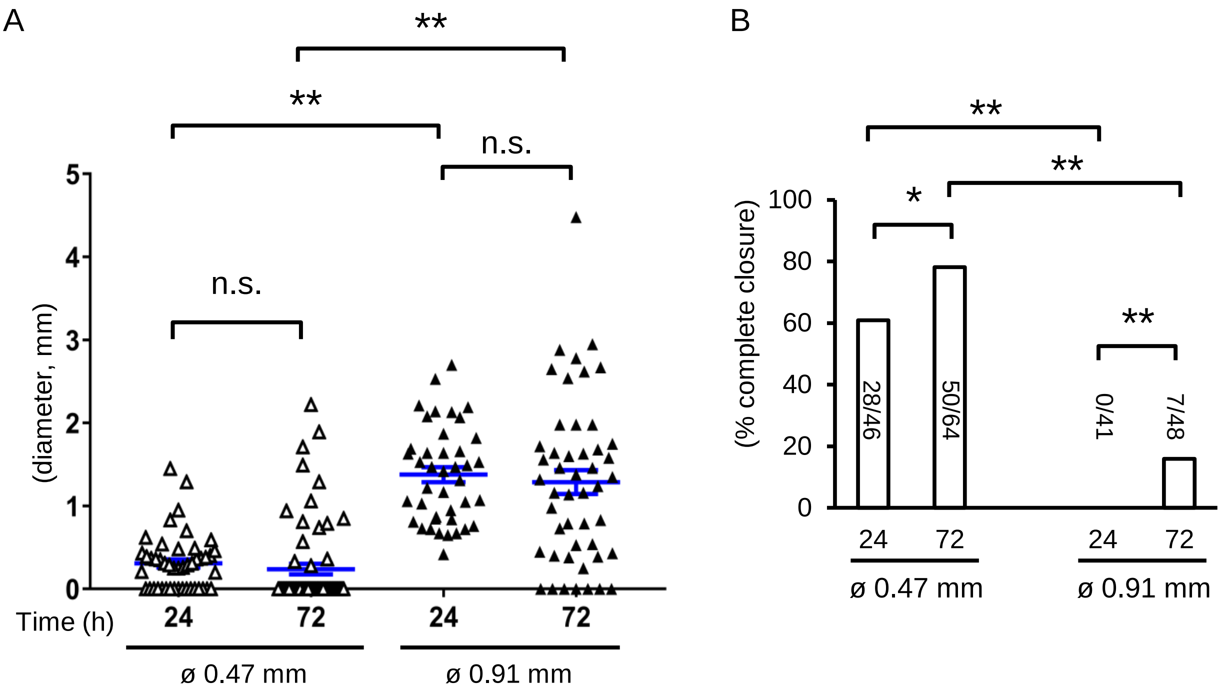

Figure S2. Perforation diameters and healing rates of choriodecidua after membrane puncture. (A) Diameter of choriodecidua with 26 G needle (ø 0.47 mm) or 20 G needle (ø 0.91 mm) at 24 and 72 h after puncture. Each symbol represents one rupture. Blue bar indicates mean and SEM. $^{**}P < 0.01$, ANOVA. **(B)** Percent complete closure of ruptured choriodecidua at 24 and 72 h. Number of completely closed ruptures/total ruptures is shown as the bar. $^{*}P < 0.05$, $^{*}P < 0.01$, χ^2. n=41-64 punctures from 12-17 fetal membranes of 3-7 pregnant mice in each group.

Figure S3

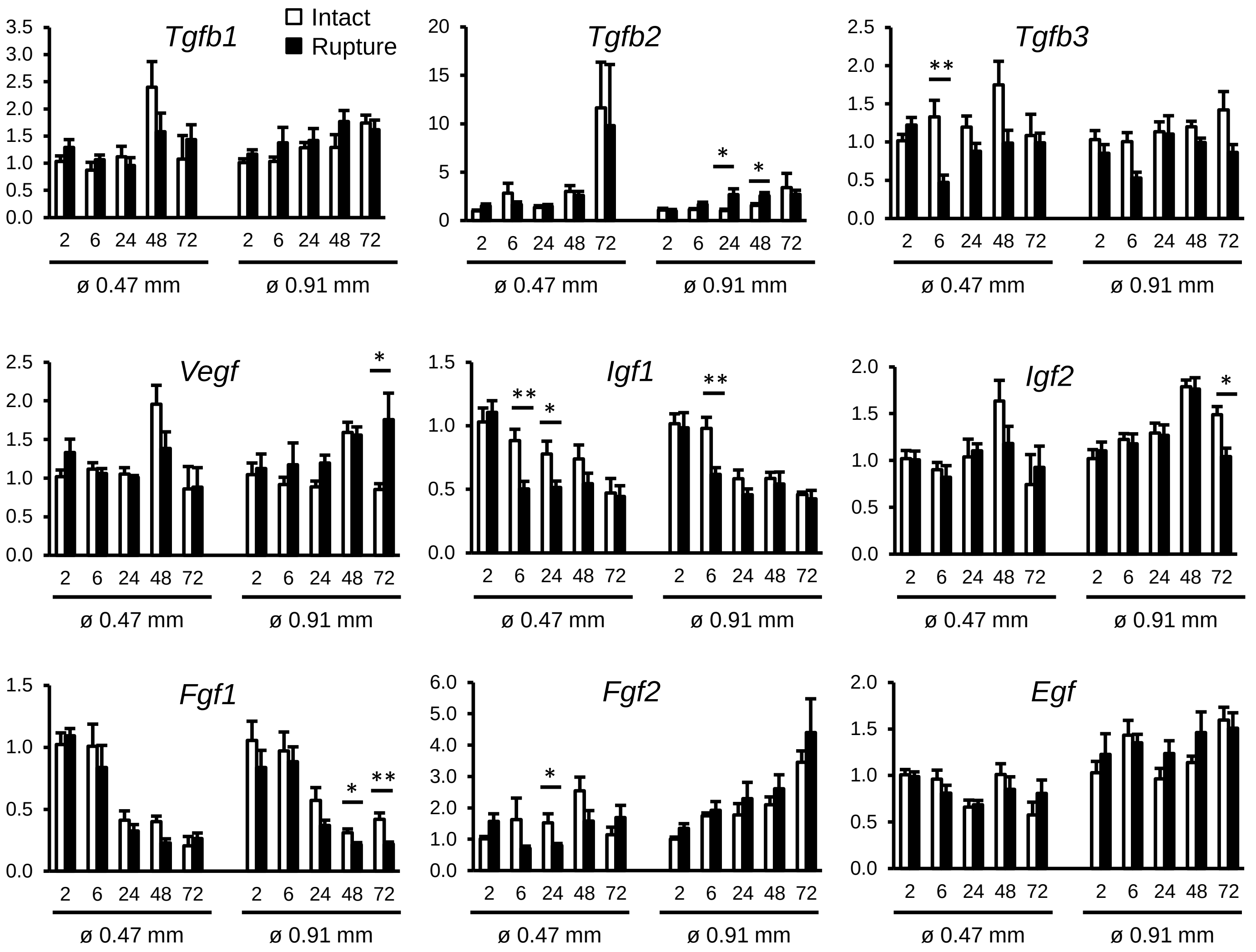

Figure S3. Expression of genes related to wound healing in ruptured fetal membrane. mRNA expression of intact or ruptured (ø 0.47 mm: 26 G needle, and ø 0.91 mm: 20 G needle) fetal membranes were analyzed by qPCR. Relative mRNA expression was compared to the level of intact membrane at 2 h. Values (relative expression) were compared at each time point. Bars, SEM. n=5-6 fetal membranes from 5-6 pregnant mice in each group. *$P < 0.05$, **$P < 0.01$.

Figure S4

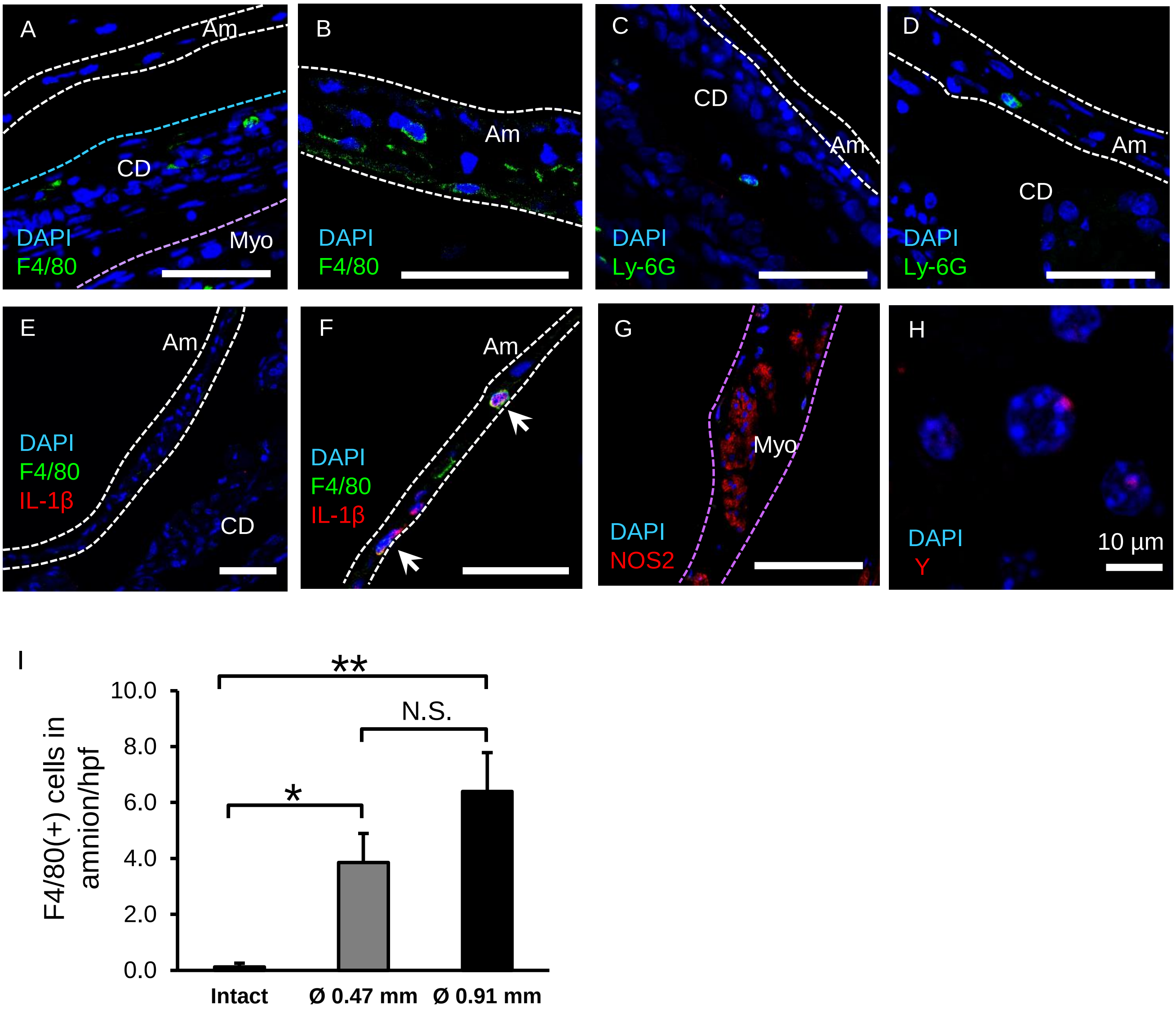

Figure S4. Macrophages at ruptured amnion. F4/80 (green) positive macrophages in intact **(A)** or healing **(B)** amnion 24 h after small rupture (ø 0.47 mm). **(C, D)** Rare Ly-6G (neutrophil marker)-positive cells in intact membrane **(C)** or healing amnion **(D)** 24 h after large rupture (ø 0.91 mm). **(E, F)** F4/80 (green) and IL-1β (red) at intact **(E)** or healing site of amnion **(F)** 24 h after small rupture (ø 0.47 mm). Arrow indicates IL-1β expressing macrophage. **(G)** NOS2 (red) in myometrium in the same slide of Figure 7A as a positive control. White dashed lines indicate amnion; purple lines denote myometrium. Bars, 50 µm. **(H)** FISH Y chromosome in male liver of adult mouse. **(I)** Number of F4/80 macrophages at the ruptured site of amnion per high powered field at 24 h. n=7-10 in each group. *$P < 0.05$, **$P < 0.01$, ANOVA**.**

Figure S5

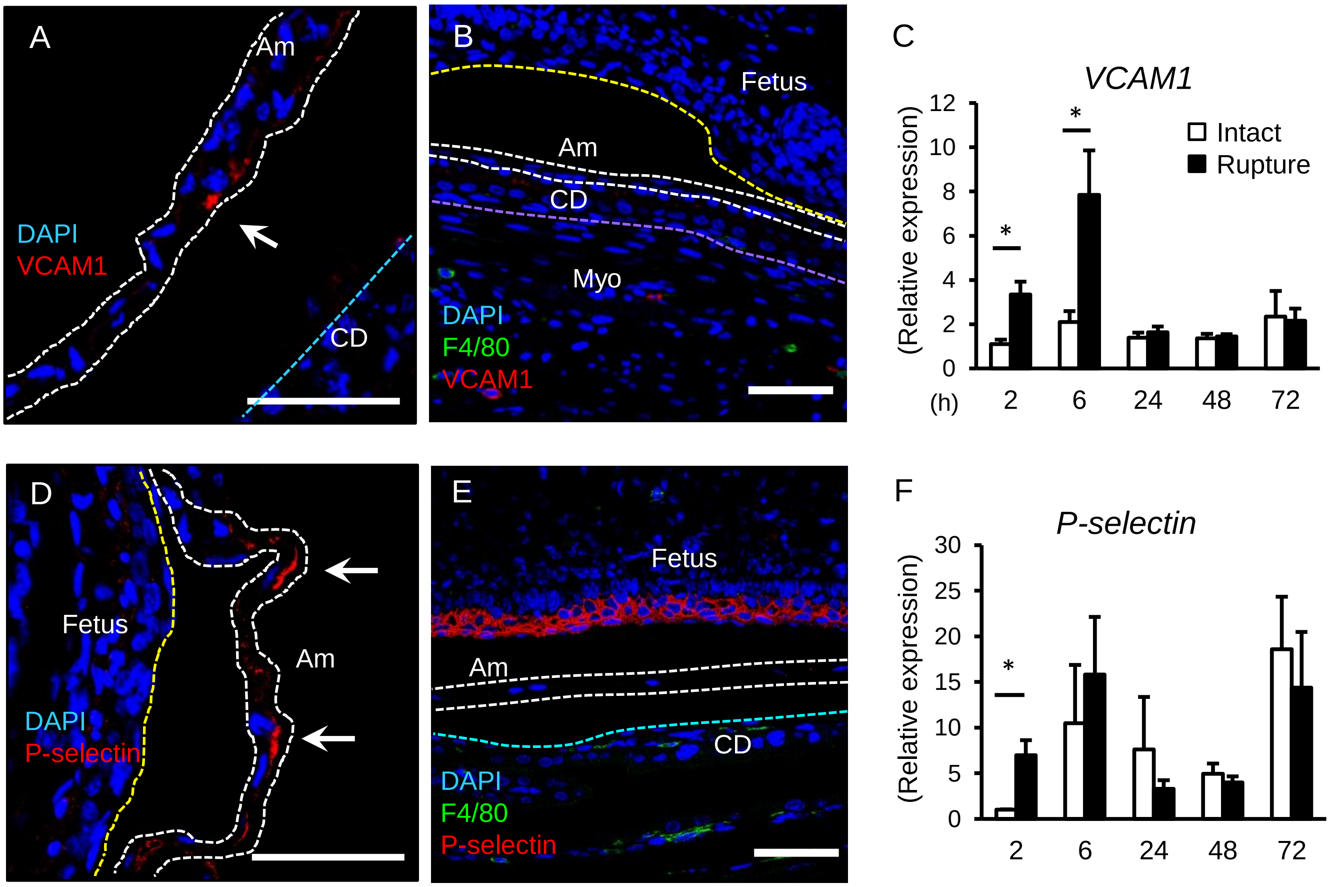

Figure S5. Macrophage adhesion molecules at ruptured amnion. Immunofluorescence (IF) staining for VCAM1 **(A and B)** or P-selectin **(D and E)** and DAPI (blue) at ruptured amnion at 24 h by 26 G needle (**A and D**, ø 0.47 mm) and intact amnion (**B and E**). Dotted lines indicate amnion (white), or surface of fetal skin (yellow). VCAM1 and P-selectin were expressed at the thicker ruptured site of amnion (arrow). Bars, 50 µm. **(C, F)** mRNA expression of *VCAM1* **(C)** and *P-selectin* **(F)**) in intact fetal membranes (open bars) or after 26 G rupture (closed bars) as a functional of time. Error bars represent SEM. n=5-6 in each group. *$P < 0.05$.

Figure S6

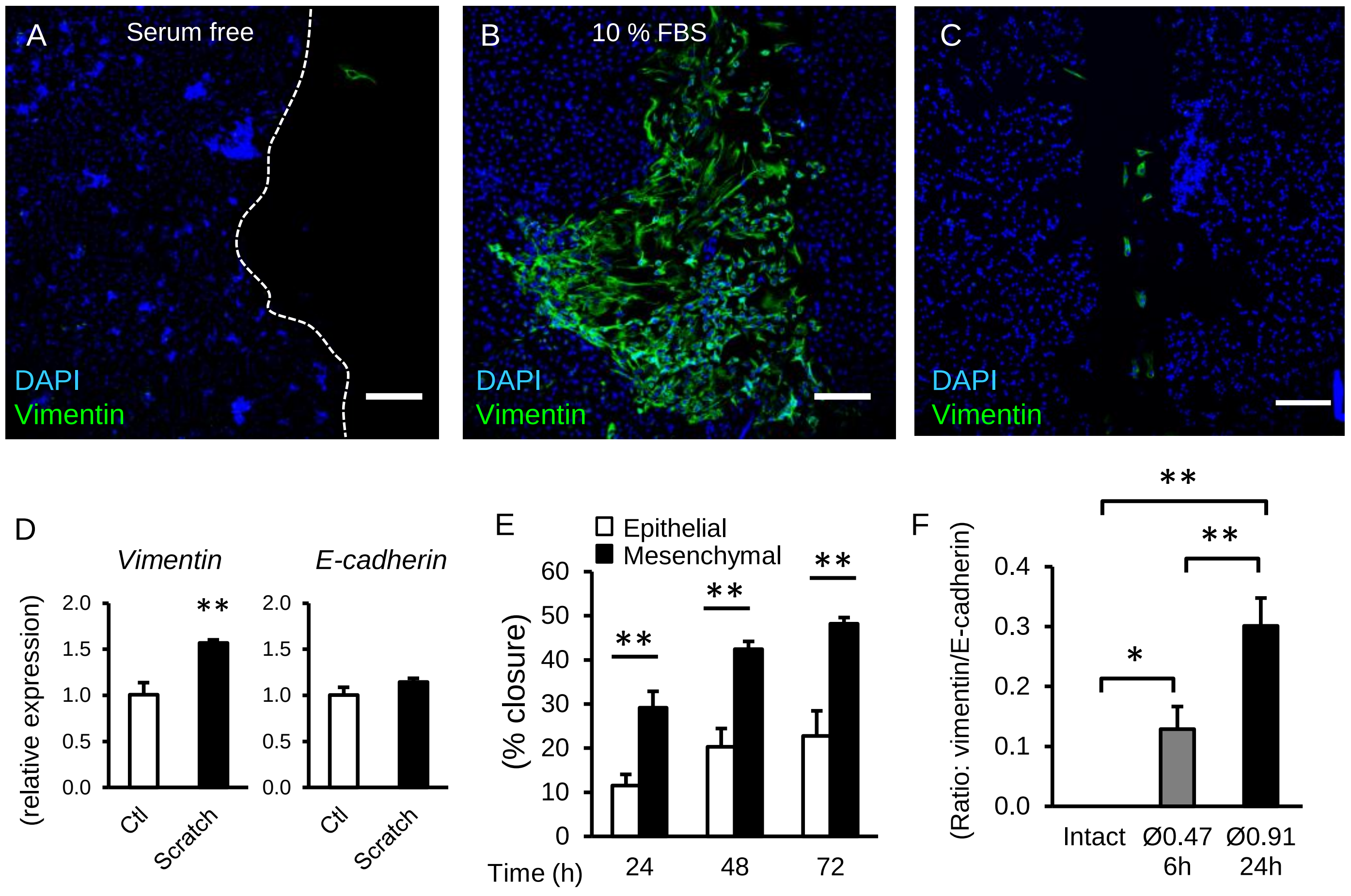

Figure S6. Epithelial-mesenchymal transition (EMT) in amnion epithelial cells. (A,B) Immunofluorescence (IF) staining for vimentin (green) and DAPI (blue) of scratched amnion epithelial cells at 8 days. Confluent primary human amnion epithelial cells were scratched, and then incubated with serum-free medium **(A)** or 10 % fetal bovine serum (FBS) medium **(B)**. Medium was changed every 2 days. **(C)** Vimentin IF of wounded epithelial cells at 5 days in serum-free medium. Bars, 200 µm. **(D)** mRNA expression of vimentin and E-cadherin in scratched amnion epithelial cells by qPCR. Confluent primary human amnion epithelial cells were scratched with a 10 µl tip and incubated for 5 days with serum-free medium. Control cells were not scratched. Gene expression was normalized to that of β2 microglobulin. Error bars represent SD. **P < 0.01. **(E)** Comparison of migratory speed between epithelial and mesenchymal cells of human amnion. Confluent primary amnion cells were scratched and percent closure compared with width at 0 h was calculated. Error bars represent SEM. n=5 (5 different placentas) in each group. ***P* < 0.01 by Student's *t*-test. **(F)** Ratio of vimentin/E-cadherin positive cells at the ruptured site of amnion in mouse model. n=4-5 in each group. **P* < 0.05, **P < 0.01, ANOVA.
